# Amuc_1473 Links Gut Microbes to Skeletal Homeostasis and Counteracts Multifactorial Osteoporosis

**DOI:** 10.1002/advs.202523067

**Published:** 2026-06-13

**Authors:** Shan‐Shan Rao, Hai‐Jin Zeng, Zun Wang, Chun‐Gu Hong, Yi‐Juan Tan, Yan‐Xin Duan, Jing‐Yao Luo, Ming‐Jie Luo, Yi‐Wei Liu, Xin Wang, Yi Luo, Teng‐Fei Wan, Yong Zhou, Zheng‐Guang Wang, Guo‐Wen Hu, Hao Yin, Xin‐Yue Hu, Zhen‐Xing Wang, Ze‐Hui He, Si‐Yi Cheng, Wei Du, Zhe Guan, Hai‐Li Lang, Hong‐Ji Liu, Jia Cao, Peng Chen, Jiang‐Hua Liu, Hui Xie, Chun‐Yuan Chen

**Affiliations:** ^1^ Department of Orthopedics Movement System Injury and Repair Research Center Xiangya Hospital Central South University Changsha Hunan China; ^2^ Hunan Key Laboratory of Angmedicine Changsha Hunan China; ^3^ National Clinical Research Center For Geriatric Disorders Xiangya Hospital Central South University Changsha Hunan China; ^4^ Experimental Center of Nursing School Xinjiang Medical University Urumqi Xinjiang China; ^5^ Department of Orthopaedics The Third Xiangya Hospital Central South University Changsha Hunan China; ^6^ Department of Neurosurgery The Second Affiliated Hospital of Nanchang University Nanchang Jiangxi China; ^7^ Department of Respiratory Medicine Xiangya Hospital Central South University Changsha Hunan China; ^8^ Department of P.E Central South University Changsha Hunan China; ^9^ Department of Rehabilitation Xiangya Hospital Central South University Changsha Hunan China; ^10^ Department of Anesthesiology The Second Affiliated Hospital of Nanchang University Nanchang Jiangxi China; ^11^ Department of Orthopaedics the First Affiliated Hospital Hengyang Medical School University of South China Hengyang China; ^12^ FuRong Laboratory Changsha Hunan China

**Keywords:** *akkermansia muciniphila*, amuc_1473, extracellular vesicles, intermittent fasting, osteoporosis

## Abstract

Emerging evidence suggests that gut microbiota‐derived signals can influence distant organs including the skeleton, yet the key microbial effectors remain elusive. Here, we identify Amuc_1473, a previously uncharacterized protein enriched in extracellular vesicles (EVs) from the commensal bacterium *Akkermansia muciniphila* (*Akk*), as a critical mediator of gut–bone communication. Amuc_1473 directly promotes osteogenesis and suppresses osteoclastogenesis by binding to negative elongation factor E (NELF‐E) and ribosomal protein L26 (RPL26), regulators of transcriptional pausing and mRNA translation, respectively. Notably, Amuc_1473 levels decline in bone and circulation under diverse pro‐osteoporotic conditions—including aging, estrogen deficiency, mechanical unloading, high‐fat diet, smoking, alcohol, and chronic stress—paralleling reductions in *Akk* and its EVs. Intermittent fasting robustly restores *Akk* abundance, Amuc_1473 levels, and bone quality in these models, via enhanced mucin production. Our findings establish Amuc_1473 as a microbial effector that systemically regulates bone homeostasis, offering a translatable strategy to prevent or treat multifactorial osteoporosis.

## Graphical abstract

Amuc_1473, a previously uncharacterized protein enriched in *Akkermansia muciniphila*‐derived extracellular vesicles, is identified as a gut–bone messenger that promotes osteogenesis and inhibits osteoclastogenesis by engaging transcriptional and translational regulators in bone cells. Its reduction across diverse osteoporotic stressors, and rescue by intermittent fasting partly via mucin induction, reveals a microbiome‐based strategy for combating multifactorial osteoporosis.

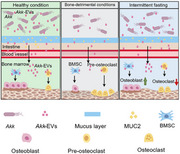

## 1 Introduction

Osteoporosis, a skeletal disorder marked by reduced bone strength and increased fracture risk [1, 2], represents a major global health burden exacerbated by aging, hormonal shifts, lifestyle, and metabolic comorbidities [3, 4, 5, 6, 7, 8]. While diverse environmental and pathological stressors—ranging from physiological and mechanical factors (e.g., aging, menopause, disuse) to dietary and lifestyle habits (e.g., high‑fat diet, alcohol intake, smoking) and various chronic diseases (e.g., diabetes, kidney disease, inflammatory disorders) [3, 4, 5, 6, 7, 8]—are known contributors, a converging mechanistic axis linking these insults to bone degeneration remains poorly defined.

Recent advances underscore the gut microbiota (GM) as a critical regulator of systemic physiology, modulating host metabolism, immunity, and even brain function [9, 10]. Notably, alterations in GM composition and its bioactive products have been implicated in the progression of bone diseases, including osteoporosis [11, 12, 13, 14, 15]. However, the key bacterial taxa and molecular mediators responsible for GM–bone communication remain largely undefined.

*Akkermansia muciniphila* (*Akk*), a mucin‐degrading bacterium that resides in the intestinal mucus layer, has emerged as a next‐generation probiotic with broad metabolic and immunological benefits [16, 17, 18]. Its reduction or depletion is commonly observed across multiple osteoporotic contexts, including menopause, aging, obesity, gut inflammation, as well as unhealthy lifestyles such as high‐fat diet, smoking, and chronic stress [18, 19, 20, 21, 22, 23, 24]. Our prior work has demonstrated that *Akk*–derived extracellular vesicles (*Akk*‐EVs) can enter bone tissue and protect against bone loss by promoting osteoblast activity and inhibiting osteoclastogenesis [17]. However, the active bone‐protective constituents of these bacterial EVs remain uncharacterized.

Here, we identify a previously unknown protein, Amuc_1473, that is highly enriched in *Akk*‐EVs and exerts potent pro‐osteogenic and anti‐osteoclastic effects. Mechanistically, Amuc_1473 interacts with NELF‐E and RPL26, modulating key transcriptional and translational pathways to enhance pro‐osteogenic β‐catenin signaling and suppress osteoclastogenesis‐related gene and protein expression. We further show that Amuc_1473 levels are consistently reduced in models of osteoporosis induced by aging, estrogen deficiency, disuse, unhealthy dietary and lifestyle factors—but can be restored through intermittent fasting, which elevates mucin levels and augments *Akk* colonization. Our findings position Amuc_1473 as a gut bacteria‐derived systemic effector that preserves host skeletal homeostasis, delineating a molecular mechanism linking the GM to bone integrity. Collectively, this study aimed to establish *Akk*‐EVs‐derived Amuc_1473 as a common therapeutic candidate for multifactorial osteoporosis, defining its molecular mechanisms, and validating microbiome‐based interventions.

## 2 Results

### 2.1 Gut *Akk* and Bone‐Localized *Akk*‐EVs are Diminished Under Multiple Osteoporotic Conditions

**FIGURE 1:**
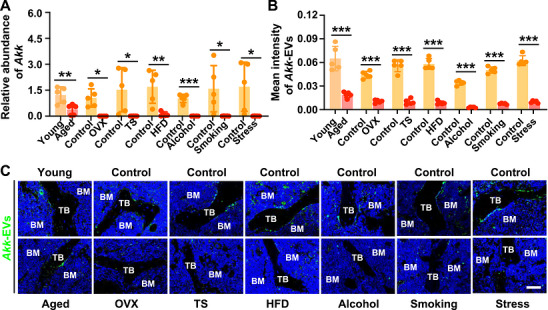
Gut *Akk* and bone‐localized *Akk*‐EVs are diminished under multiple osteoporotic conditions. (A) qRT‐PCR analysis of gut *Akk* abundance in young (3‐month‐old) and aged (18‐month‐old) mice, or in 3‐month‐old young mice exposed to different bone‐detrimental factors, including ovariectomy (OVX), tail suspension (TS), high‐fat diet (HFD), alcohol intake, smoking, and chronic stress. *n* = 5 per group. (B,C) Quantification of mean staining intensity (B) and representative images (C) of *Akk*‐EVs‐stained bone sections from control and different bone‐detrimental factors‐treated mice. Scale bar: 50 µm. Data are mean ± SD. Statistics: unpaired, two‐tailed Student's *t*‐test (A,B). ^*^ *p* < 0.05, ^**^ *p* < 0.01, ^***^ *p* < 0.001.

To examine the impact of aging on the abundance of *Akk*, we used C57BL/6J mice, a strain that exhibits marked age‐related bone loss compared with other genotypes [25]. We collected fecal microbiota from young (3‐month‐old) and aged (18‐month‐old) C57BL/6J mice and performed quantitative reverse transcription polymerase chain reaction (qRT‐PCR) analysis. As shown in Figure 1A, gut *Akk* levels were significantly reduced in aged mice compared to young controls, consistent with our previous observations in humans [17].

We next established a series of osteoporosis models in young C57BL/6J mice by exposing them to a range of bone‐detrimental stimuli, including estrogen deficiency via ovariectomy (a well‐established model of postmenopausal osteoporosis), mechanical unloading using tail suspension (TS, mimicking disuse‐induced osteoporosis), high‐fat diet (HFD), alcohol consumption, cigarette smoke exposure, and chronic psychological stress. qRT‐PCR analysis revealed that all these interventions led to significant reductions in fecal *Akk* abundance (Figure 1A).

To determine whether the decrease in *Akk* was associated with altered delivery of bacterial EVs to the bone, we assessed *Akk*‐EVs in the skeletal tissue of treated mice. Immunofluorescence staining revealed strong *Akk*‐EVs signals on the trabecular bone surfaces and within the bone marrow compartments of control animals (Figure 1B,C). In contrast, mice subjected to the above bone‐damaging conditions exhibited markedly diminished *Akk*‐EV signals (Figure 1B,C), suggesting that suppression of gut *Akk* by diverse pathological factors impairs the transfer of its EVs to the bone.

### 2.2 *Akk*‐EVs‐Enriched Amuc_1473 Protein Displays Robust Pro‐Osteogenic and Anti‐Osteoclastic Activity

**FIGURE 2:**
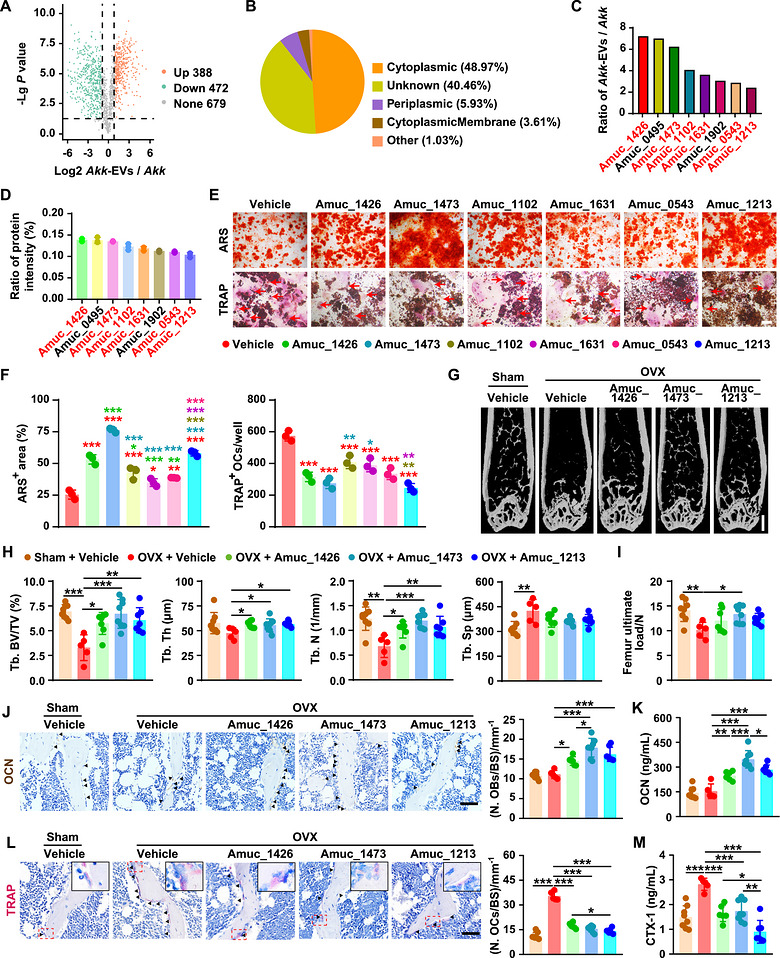
Akk‐EVs‐enriched Amuc_1473 protein displays robust pro‐osteogenic and anti‐osteoclastic activity. (A) Volcano plot showing differentially expressed proteins (|fold change| ≥ 2; *P* < 0.05) in *Akk*‐EVs vs. *Akk*. (B) Subcellular localization of up‐regulated proteins in *Akk*‐EVs. (C,D) Expression ratios (C) and relative abundance (D) of candidate proteins in *Akk*‐EVs (>0.1% of total protein). (E) Alizarin red S (ARS) and tartrate resistant acid phosphatase (TRAP) staining images in BMSCs and RAW264.7 cells under various treatments. Scale bar: 200 µm. (F) Quantification of ARS^+^ area and osteoclast numbers. *n* = 3 per group. (G) µCT images of distal femurs from sham or OVX mice with different treatments. Scale bars: 1 mm. (H) Quantification of trabecular bone volume fraction (Tb. BV/TV), thickness (Tb. Th), number (Tb. N), and separation (Tb. Sp). *n* = 8 (Sham + Vehicle or OVX + Amuc_1473), 5 (OVX + Vehicle), or 7 (OVX + Amuc_1426 or OVX + Amuc_1213). (I) Femur ultimate load by three‐point bending test. *n* = 5–8 per group. (J) Osteocalcin (OCN) staining images with quantification of osteoblast number on trabecular bone surface (N. OBs/BS). *n* = 5–8 per group. Scale bar: 50 µm. (K) ELISA for serum OCN. *n* = 5–8 per group. (L,M) TRAP staining images with quantification of osteoclast number (N. OCs) (L) and ELISA for serum C‐terminal telopeptides of type I collagen (CTX‐1). *n* = 5–8 per group. Scale bar: 50 µm. Data are mean ± SD. Statistics: unpaired, two‐tailed Student's *t*‐test (A; H–M: OVX + Vehicle vs. Sham + Vehicle); one‐way ANOVA with Bonferroni post hoc test (F; H–M: comparisons among OVX groups). ^*^ *p* < 0.05, ^**^ *p* < 0.01, ^***^ *p* < 0.001.

To identify active components mediating the skeletal benefits of *Akk*‐EVs, we conducted quantitative proteomic profiling of *Akk*‐EVs and their parent bacterium. Among 1685 identified proteins, 1539 were reliably quantified, revealing 860 differentially expressed candidates (|fold change| ≥ 2; *p* < 0.05). Of these, 388 were significantly enriched in *Akk*‐EVs relative to whole‐cell lysates (Figure 2A and Table S1). Gene ontology analysis indicated that the majority of these proteins originated from cytoplasmic or unknown compartments (Figure 2B), with functional classification implicating roles in metabolic regulation, energy production, and biosynthetic pathways (Table S2).

From this pool, we first excluded proteins mainly involved in basic bacterial structure, defense, and transport, as well as highly conserved and periplasmic proteins, to avoid generic housekeeping components and emphasize EV‐specific factors. We then selected proteins whose intensity in *Akk*‐EVs exceeded 0.1% of the total EV proteome, yielding eight *Akk*‐EVs–enriched candidates (Amuc_1426, Amuc_0495, Amuc_1473, Amuc_1102, Amuc_1631, Amuc_1902, Amuc_0543, and Amuc_1213) for further evaluation (Figure 2C,D). Notably, Amuc_1631 (also known as P9) has previously been implicated in enhancing glucose homeostasis and ameliorating metabolic dysfunction in mice [26]. Amuc_0495 and Amuc_1902 were subsequently excluded from further analysis due to technical failure in recombinant expression or protein purification.

To assess functional relevance, we examined the effects of the remaining six proteins on osteogenic differentiation of mouse primary bone marrow mesenchymal stromal cells (BMSCs) and osteoclast formation from RAW264.7 macrophages. BMSCs exhibited short spindle‐shaped or irregular morphology, possessed multi‐lineage differentiation potential into osteogenic, adipogenic, and chondrogenic lineages, and expressed typical MSC markers (Sca‐1, CD29, CD44, and CD90), but lacked hematopoietic/endothelial markers (CD31 and CD45) (Figure S1A–C). RAW264.7 cell showed minimal spontaneous differentiation under un‐induced condition (Figure S1D). Remarkably, as shown by Alizarin Red S (ARS) and tartrate‐resistant acid phosphatase (TRAP) staining, all candidates promoted mineralized matrix deposition of BMSCs and suppressed osteoclast formation of RAW264.7 cells (Figure 2E,F). Among them, Amuc_1473 exhibited the strongest osteo‐anabolic effect and the second most potent anti‐osteoclastic activity, while Amuc_1213 was most effective in suppressing osteoclast formation (Figure 2E,F). Amuc_1426 showed the third highest pro‐osteogenic and anti‐osteoclastic effects (Figure 2E,F). Based on this dual functionality, these three proteins were selected for in vivo validation.

In 3‐month‐old ovariectomized (OVX) mice, weekly systemic administration of these proteins for 8 weeks could not reverse the reduction of uterus sizes and weights (Figure S2A,B), and did not notably affect the levels of liver and kidney function‐related markers (Figure S2C,D), indicating a lack of obvious hepatotoxicity or nephrotoxicity. However, these bacterial proteins significantly reversed the osteoporotic phenotypes induced by OVX. µCT analyses of the femur, tibia, and lumbar vertebrae (L3–L4) revealed significantly improved trabecular bone volume (Tb. BV/TV), thickness (Tb. Th), and/or number (Tb. N), along with reduced separation (Tb. Sp), across all three treatment groups (Figure 2G,H, and Figure S3A–D). Notably, Amuc_1473 exerted the greatest overall bone structural restoration and was the only protein to significantly enhance femoral strength in biomechanical testing (Figure 2G–I and Figure S3A–D). Histological and biochemical analyses further corroborated the superior activity of Amuc_1473. It most strongly increased osteocalcin (OCN)‐positive osteoblasts and serum OCN levels (Figure 2J,K), consistent with the highest pro‐osteogenic effect in vitro. Although Amuc_1213 remained the most effective in inhibiting osteoclasts and reducing serum C‐terminal telopeptides of type I collagen (CTX‐1), Amuc_1413 exhibited balanced and robust effects on both bone formation and resorption (Figure 2J–M). Together, these findings establish Amuc_1473 as the most potent *Akk*‐EV‐enriched protein in modulating bone remodeling, combining osteo‐anabolic and anti‐resorptive activities in vitro and in vivo. This dual capacity prompted its selection for downstream mechanistic investigation.

Since increased gut barrier permeability promotes gut‐to‐circulation translocation of bacterial products [27] and contributes to estrogen deficiency‐induced osteoporosis [28], we examined the effects of three *Akk*‐EV‐enriched proteins (Amuc_1473, Amuc_1213, and Amuc_1426) on intestinal barrier function. qRT‐PCR analysis of intestinal tissues revealed that both Amuc_1473 and Amuc_1213 markedly upregulated the mRNA levels of tight‐junction protein *Claudin‐1* (*Cldn1*) and downregulated pore‐forming protein *Cldn15* (Figure S4A). Amuc_1473 further increased the expression of *Cldn4*, junctional adhesion molecule 3 (*Jam3*), and *Occludin* (*Ocln*), while downregulating the levels of *Cldn2* (Figure S4A). ELISA assays showed significant reductions in serum lipopolysaccharide (LPS; endotoxin) and Zonulin‐1 levels after treatment with these three proteins in OVX mice, with Amuc_1473 showing a trend toward more pronounced effects (Figure S4B). These results indicate that these *Akk*‐EVs‐enriched proteins, particularly Amuc_1473, reinforce rather than disrupt gut epithelial integrity, which might be a mechanism contributing to their bone‐protective effects.

### 2.3 Amuc_1473 Levels Decline in Bone‐Detrimental States and Positively Correlates With Bone Formation and Mass

**FIGURE 3:**
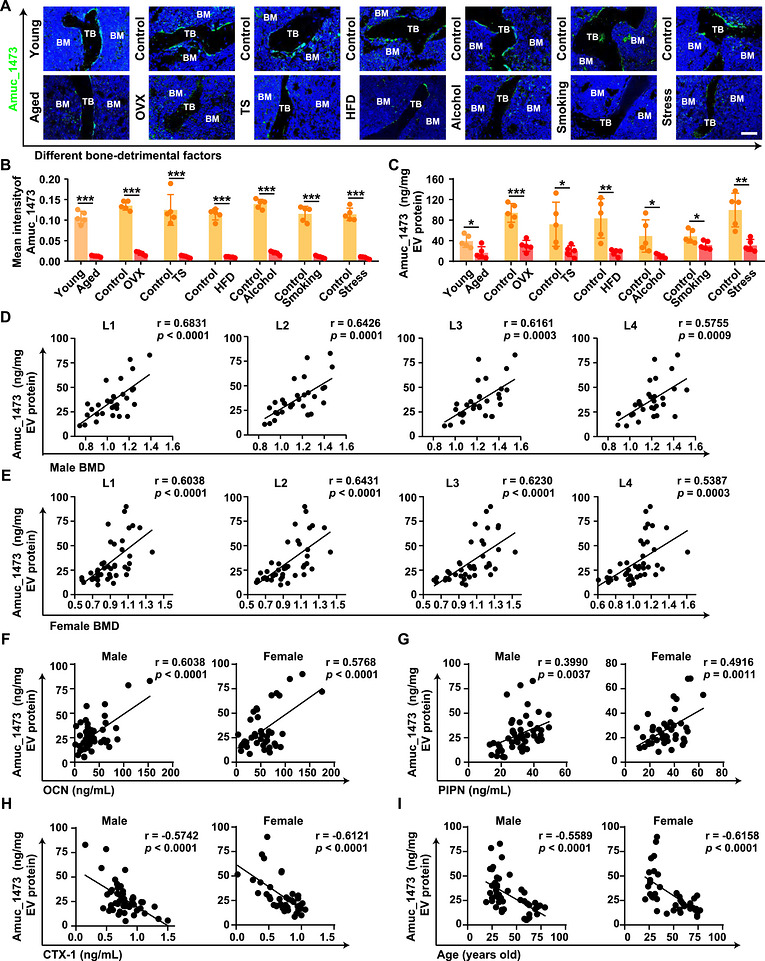
Amuc_1473 declines in bone‐detrimental states and positively correlates with bone formation and mass. (A,B) Amuc_1473 staining images (A) and quantification of the mean intensity (B) in distal femurs from control and different bone‐detrimental factors‐treated mice. Scale bar: 50 µm. *n* = 5 per group. (C) ELISA for Amuc_1473 in plasma EVs from control mice or those exposed to different bone‐detrimental factors. *n* = 5 per group. (D,E) Correlation of Amuc_1473 level in human plasma EVs with bone mineral intensity (BMD) in the L1 to L4 lumbar vertebra. *n* = 30 (male) or 41 (female) per group. (F–H) Correlation of Amuc_1473 level in human plasma EVs with the concentrations of markers related to bone formation (OCN and PINP; F and G) or bone resorption (CTX‐1; H) in serum. PINP: procollagen type I N‐terminal propeptide. *n* = 51 (male) or 41 (female) per group. (I) Correlation of Amuc_1473 level in human plasma EVs with the age of donors. *n* = 51 (male) or 41 (female) per group. Data are mean ± SD. Statistics: unpaired, two‐tailed Student's *t*‐test (B,C); two‐tailed Pearson's Correlation analysis (D–I). ^*^ *p* < 0.05, ^**^ *p* < 0.01, ^***^ *p* < 0.001.

To determine whether the levels of Amuc_1473 are affected by bone‐damaging stimuli, we generated a custom antibody and performed immunofluorescence staining on femoral sections from mice subjected to various pro‐osteoporotic conditions. In young healthy controls, Amuc_1473 signals were readily detected on trabecular bone surfaces and within bone marrow compartments (Figure 3A,B), confirming that the protein is transported from the gut to the bone under physiological conditions.

In contrast, Amuc_1473 expression was markedly reduced in all pathological models examined, including aging, OVX‐induced estrogen deficiency, tail suspension–induced hind‐limb disuse, high‐fat diet, ethanol exposure, cigarette smoke inhalation, and chronic stress (Figure 3A,B). These reductions mirrored the declines in gut *Akk* and bone‐localized *Akk*‐EVs, suggesting that Amuc_1473 is a downstream effector sensitive to diverse bone‐detrimental factors. Consistent with these findings, ELISA assays showed that circulating Amuc_1473 levels in plasma EVs were also significantly diminished across all pro‐osteoporotic conditions (Figure 3C).

To investigate the translational relevance of these observations, we measured Amuc_1473 levels in plasma EVs from a human donor cohort spanning a wide age range (male: 19–79 years; female: 21–77 years) and examined their associations with bone health parameters. Pearson correlation analyses revealed a strong positive correlation between plasma Amuc_1473 abundance and bone mineral density (BMD) in both sexes (Figure 3D,E), as well as with serum levels of bone formation markers including OCN and procollagen type I N‐terminal propeptide (PINP) (Figure 3F,G). Conversely, Amuc_1473 levels in plasma EVs were inversely correlated with the bone resorption marker CTX‐I (Figure 3H) and with chronological age (Figure 3I). In humans with osteopenia or osteoporosis, Amuc_1473 levels were markedly reduced compared with those having normal bone mass, with the lowest levels in the osteoporosis group (Figure S5A). We stratified the male and female plasma EV samples into three age groups (19–44 years, 45–60 years, and >60 years) and compared Amuc_1473 levels across these groups. No significant sex differences were observed within these age strata (Figure S5B).

Together, these findings support the notion that Amuc_1473 functions as a systemic effector linking gut microbial activity to bone metabolic status, and that its loss underlies the skeletal dysregulation observed in multifactorial osteoporosis.

### 2.4 Amuc_1473 Drive Osteogenesis via Binding to NELF‐E and Repressing PSME2

**FIGURE 4:**
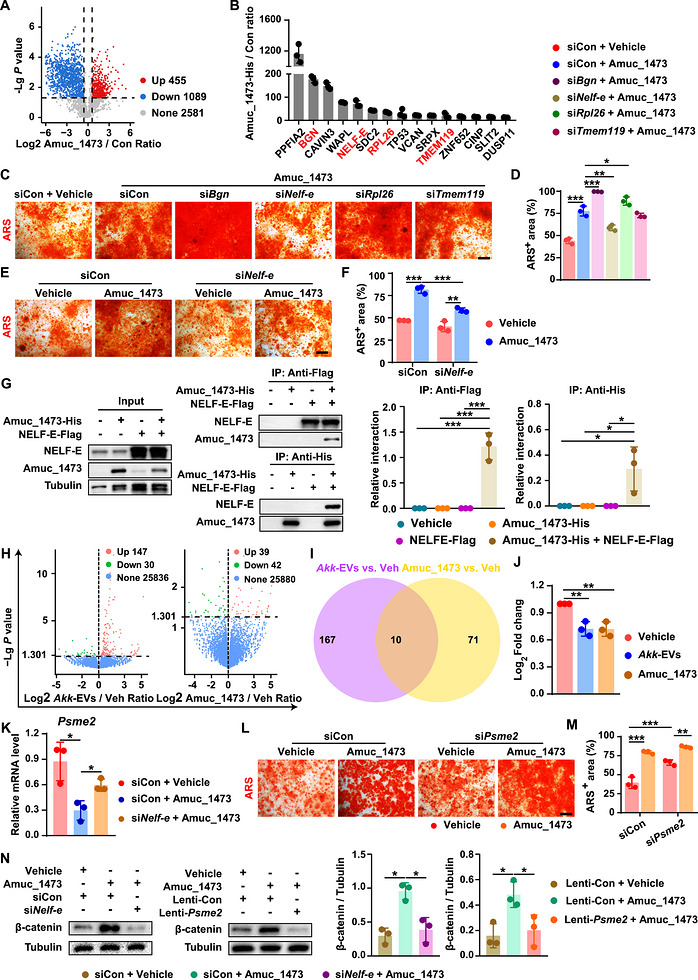
Amuc_1473 drive osteogenesis via binding to NELF‐E and repressing PSME2. (A) Volcano plot showing proteins differentially pulled down by Amuc_1473‐His + anti‐His‐conjugated beads (Amuc_1473‐His group) vs. anti‐His‐beads alone (control group) in BMSCs under osteogenic induction (|fold change| ≥ 1.5 and *p* < 0.05). *n* = 3 per group. (B) Expression ratios of top 15 enriched proteins in Amuc_1473‐His vs. control group. *n* = 3 per group. (C–F) ARS staining images (C,E) and quantification of ARS^+^ areas (D,F) in BMSCs treated with different siRNAs ± Amuc_1473 under osteogenic induction. Scale bar: 200 µm. *n* = 3 per group. (G) Co‐immunoprecipitation of Amuc_1473‐His with NELF‐E‐Flag in transfected Chinese hamster ovary (CHO) cells. *n* = 3 per group. (H) Volcano plot of differentially expressed genes (DEGs; |log_2_(foldchange)| > 0.0 and *p* < 0.05) in BMSCs receiving different treatments under osteogenic condition. Veh: vehicle. *n* = 3 per group. (I) Venn diagram of DEG overlap between the two comparisons. (J,K) *Psme2* mRNA levels in BMSCs with different treatments assessed by transcriptome sequencing (J) or qRT‐PCR (K). *n* = 3 per group. (L,M) ARS staining images (L) and quantification of ARS^+^ area ratios (M) in BMSCs treated with different siRNAs ± Amuc_1473 under osteogenic differentiation. Scale bar: 200 µm. *n* = 3 per group. (N) Western blotting for β‐catenin protein in BMSCs with different treatments. *n* = 3 per group. Data are mean ± SD or mean ± SEM. Statistics: unpaired two‐tailed Student's *t*‐test (A; D: siCon + Vehicle vs. siCon + Amuc_1473; H,K,N); one‐way ANOVA with Bonferroni post hoc test (D: comparisons among siRNA + Amuc_1473 groups; G,J); or two‐way ANOVA with Bonferroni post hoc test (F,M). ^*^ *p* < 0.05, ^**^ *p* < 0.01, ^***^ *p* < 0.001.

To elucidate the molecular mechanism through which Amuc_1473 directly promotes osteogenesis, we first sought to identify its intracellular binding partners. BMSCs were treated with His‐tagged Amuc_1473 under osteogenic conditions, followed by a pull‐down assay using anti‐His–conjugated agarose beads and proteomic analysis. Cells treated with beads alone served as controls. A total of 4125 proteins were quantified, among which 1544 were differentially expressed (|fold change| ≥ 1.5, *p* < 0.05), including 455 proteins enriched in Amuc_1473‐His group (Figure 4A and Table S3).

Among the top fifteen most abundant proteins in Amuc_1473‐His group compared with controls (Figure 4B), we focused on four proteins previously implicated in osteogenesis—biglycan (BGN), negative elongation factor E (NELF‐E), ribosomal protein L26 (RPL26), and transmembrane protein 119 (TMEM119) [29, 30, 31, 32]. ARS staining showed that, among siRNAs targeting these genes, only *Nelf‐e* knockdown significantly impaired the pro‐osteogenic effect of Amuc_1473 (Figure 4C–F), identifying NELF‐E as a critical downstream mediator of Amuc_1473‐driven osteogenesis.

We next validated the physical interaction between Amuc_1473 and NELF‐E. Chinese hamster ovary (CHO) cells expressing Flag‐tagged NELF‐E were treated with His‐tagged Amuc_1473, followed by co‐immunoprecipitation by anti‐Flag or anti‐His. Western blotting confirmed reciprocal binding: Amuc_1473 was detected in anti‐Flag immunoprecipitates and NELF‐E in anti‐His pull‐downs (Figure 4G), supporting the binding relationship between these two proteins. A faint band was observed in the input sample from cells trasfected with NELF‐E‐Flag alone at a molecular weight close to that of Amuc_1473 in Figure 4G, which likely resulted from residual NELF‐E signal after sequential membrane probing rather than specific Amuc_1473 detection.

Given that NELF‐E has been characterized as a transcriptional suppressor [33], we hypothesized that Amuc_1473 may function NELF‐E to regulate gene expression. Transcriptome sequencing of BMSCs treated with vehicle, *Akk*‐EVs, or Amuc_1473 identified 147 genes up‐regulated and 30 down‐regulated in *Ak*k‐EVs group, and 39 up‐regulated and 42 down‐regulated in Amuc_1473 group, relative to controls (Figure 4H and Table S4). Notably, ten differentially expressed genes overlapped between the two treatments (Figure 4I). Among them, *Psme2*, a known inhibitor of osteogenesis [34], was consistently downregulated by both *Akk*‐EVs and Amuc_1473 (Figure 4J).

To test whether the negative effect of Amuc_1473 on *Psme2* expression was dependent on NELF‐E, we silenced *Nelf‐e* in BMSCs and observed that Amuc_1473‐induced *Psme2* repression was profoundly reversed (Figure 4K), confirming *Psme2* as a transcriptional target of the Amuc_1473–NELF‐E axis. Functionally, knockdown of *Psme2* downregulated the pro‐osteogenic effect by Amuc_1473 (Figure 4L,M), indicating that *Psme2* suppression is required for Amuc_1473‐mediated promotion of osteogenesis. Wnt/β‐catenin signaling, a key driver of osteogenesis, is negatively regulated by PSME2 and NELF‐E [30, 34, 35]. Consistently, Amuc_1473 treatment elevated β‐catenin protein levels in BMSCs, but this effect was blunted by either *Nelf‐e* knockdown or *Psme2* overexpression (Figure 4N). Immunofluorescence staining revealed that Amuc_1473 promoted the translocation of β‐catenin from the cytoplasm into the nucleus (Figure S6A). Consistently, western blotting showed that Amuc_1473 reduced the levels of phosphorylated β‐catenin while increasing the level of total β‑catenin (Figure S6B,C), indicative of increased stabilization of β‐catenin and activation of the Wnt/β‑catenin pathway. Furthermore, knockdown of *Nelf‐e* or *Psme2* overexpression abolished the Amuc_1473‐induced changes in β‐catenin nuclear localization and phosphorylation (Figure S6A–C), supporting a critical role of NELF‑E and PSME2 in Amuc_1473‐mediated activation of Wnt/β‐catenin signaling. Collectively, these results define a mechanistic cascade wherein Amuc_1473 binds to NELF‐E to transcriptionally repress *Psme2* expression, thereby releasing Wnt/β‐catenin signaling and promoting osteogenic differentiation.

### 2.5 Amuc_1473 Suppresses Osteoclastogenesis via NELF‐E‐Mediated *Fcgr3* Repression and RPL26‐Driven EGLN2 Translation

**FIGURE 5:**
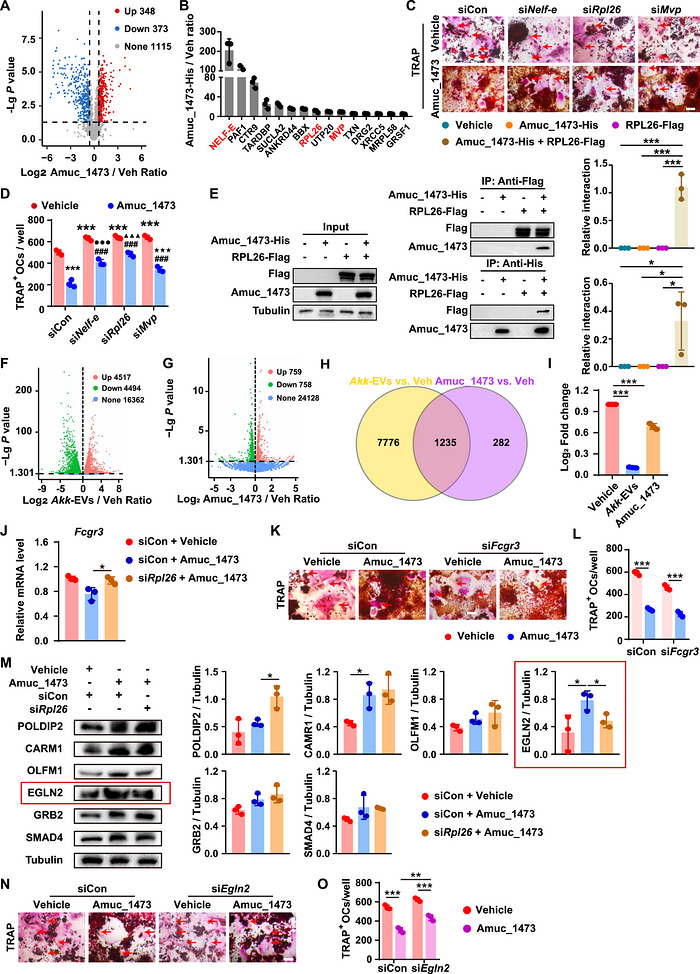
Amuc_1473 suppresses osteoclastogenesis via NELF‐E‐mediated *Fcgr3* repression and RPL26‐driven EGLN2 translation. (A) Volcano plot showing proteins differentially pulled down by Amuc_1473‐His vs. control beads in osteoclast‐differentiating RAW264.7 cells (|fold change| ≥ 1.5 and *P* < 0.05). *n* = 3 per group. (B) Expression ratios of top 15 enriched proteins in Amuc_1473‐His vs. controls. *n* = 3 per group. (C,D) TRAP staining (C) and osteoclast quantification (D) in RAW264.7 treated with different siRNAs ± Amuc_1473. Scale bar: 200 µm. *n* = 3 per group. ^***^ *P* < 0.001 vs. siCon + Vehicle; ^###^ *P* < 0.001 vs. siCon + Amuc_1473; ^●●●^ *p* < 0.01 vs. si*Nelf‐e* + Vehicle; ^▲▲▲^ *p* < 0.05 vs. si*Rpl26* + Vehicle; ^★★★^ *p* < 0.05 vs. si*Mvp* + Vehicle. (E) Co‐immunoprecipitation of Amuc_1473‐His with RPL26‐Flag in transfected CHO cells. (F,G) Volcano plot showing DEGs (|log_2_(foldchange)| > 0.0 and *p* < 0.05) in RAW264.7 receiving different treatments. *n* = 3 per group. (H) Venn diagram of DEG overlap between the two comparisons. (I, J) *Fcgr3* mRNA levels in RAW264.7 with different treatments assessed by transcriptome sequencing (I) or qRT‐PCR (J). *n* = 3 per group. (K,L) TRAP staining (K) and osteoclast quantification (L) in RAW264.7 receiving different treatments. Scale bar: 200 µm. *n* = 3 per group. (M) Western blotting for potential downstream effectors of RPL26. (N,O) TRAP staining (N) and osteoclast quantification (O) in RAW264.7 with different treatments. Scale bar: 200 µm. *n* = 3 per group. Data are mean ± SD or mean ± SEM. Statistics: unpaired, two‐tailed Student's *t*‐test (A,F,G,J,M); two‐way ANOVA with Bonferroni post hoc test (D,L,O); one‐way ANOVA with Bonferroni post hoc test (E, I). ^*^ *p* < 0.05, ^**^ *p* < 0.01, ^***^ *p* < 0.001.

To elucidate the molecular mechanism underlying the anti‐osteoclastic activity of Amuc_1473, we conducted His‐tag pull‐down assays in RAW264.7 cells under osteoclastic induction, followed by proteomic analysis. A total of 1836 proteins were quantified, among which 721 were differentially enriched in Amuc_1473‐His–treated cells compared to controls (|fold change| ≥ 1.5, *p* < 0.05) (Figure 5A and Table S5). Specifically, 348 proteins were up‐regulated, and 373 were down‐regulated in Amuc_1473‐His group (Figure 5A and Table S5).

From the top 15 enriched proteins (Figure 5B), we prioritized three candidates—major vault protein (MVP), NELF‐E, and RPL26—for further investigation, based on their reported involvement in transcriptional repression, translational regulation, or osteoclastogenesis [32, 33, 36]. TRAP staining showed that siRNA‐mediated knockdown of these genes increased osteoclast formation and markedly blocked the anti‐osteoclastic effect of Amuc_1473, while si*Nelf‐e* and si*Rpl26* more potently than si*Mvp* impaired the anti‐osteoclastic effect of Amuc_1473 (Figure 5C,D). Thus, NELF‐E and RPL26 were selected for subsequent mechanistic validation. Anti‐His pull‐down assays using purified recombinant proteins showed efficient pull down of NELF‐E from the NELF‐E/Amuc_1473‐His complex (Figure S7A), confirming direct binding of Amuc_1473 to NELF‐E in vitro. Mirroring the interaction between Amuc_1473 and NELF‐E in Figure 4G, co‐immunoprecipitation confirmed the physical interaction between Amuc_1473 and RPL26, which showed that His‐tagged Amuc_1473‐His co‐precipitated with RPL26‐Flag in CHO cells using either anti‐His or anti‐Flag antibodies (Figure 5E). These findings suggest that Amuc_1473 can concurrently engage both NELF‐E and RPL26 to regulate osteoclastogenesis.

Furthermore, immunofluorescence staining and co‐immunoprecipitation assays were performed to confirm their interactions in vivo. Immunofluorescence staining of femurs from 5‐month‐old adult mice revealed abundant co‐localization signals between Amuc_1473 and both NELF‐E and RPL26 (Figure S7B). Co‐immunoprecipitation of bone lysates from these mice using anti‐NELF‐E or anti‐RPL26 antibodies (vs. IgG controls) specifically pulled down Amuc_1473, whereas IgG controls showed minimal non‐specific binding (Figure S7C). These results demonstrate that *Akk*‐EVs‐enriched Amuc_1473 reaches bone tissue and associates with NELF‐E and RPL26 under physiological conditions.

Given the established role of NELF‐E in repressing gene transcription [37, 38], we next asked whether it mediates Amuc_1473‐driven suppression of osteoclastogenesis‐related gene expression in RAW264.7 cells. Transcriptome profiling identified 9011 differentially expressed genes in *Akk*‐EVs‐treated group and 1,517 in Amuc_1473‐treated cells relative to the vehicle‐treated controls (|log_2_(fold change)| > 0.0, *p* < 0.05) (Figure 5F,G and Table S6). Notably, 1235 genes overlapped between the two datasets (Figure 5H). Among them, *Fcgr3*, a known promoter of osteoclast differentiation [32, 33, 36], was significantly down‐regulated by both *Akk*‐EVs and Amuc_1473 (Figure 5I). This suppression was validated by qRT‐PCR and was reversed upon *Nelf‐e* knockdown, confirming NELF‐E–dependent repression of *Fcgr3* (Figure 5J). Functionally, both *Fcgr3* siRNA and Amuc_1473 individually inhibited osteoclast formation, and the anti‐osteoclastic effect of Amuc_1473 was attenuated but not abolished when *Fcgr3* was inhibited (Figure 5K,L), indicating that *Fcgr3* repression is a partial contributor to Amuc_1473‐mediated osteoclast inhibition.

To investigate the contribution of RPL26, a regulator of RNA translation [32, 36], we employed catRAPID omics v2.1 to predict mRNAs with high affinity for RPL26 (Table S7). Among the top‐ranked candidates, several encode proteins have been shown to exhibit anti‐osteoclastic roles, including POLDIP2, CARM1, OLFM1, EGLN2, GRB2, and SMAD4 [39, 40, 41, 42, 43]. Western blotting showed that Amuc_1473 treatment increased the expression of multiple candidates under osteoclastic conditions, with EGLN2 being the only protein whose upregulation was significantly impaired by *Rpl26* knockdown (Figure 5M). TRAP staining showed that *Egln2* suppression remarkably enhanced osteoclast formation and counteracted the anti‐osteoclastic effect of Amuc_1473 (Figure 5N,O), establishing EGLN2 as a critical downstream effector in the RPL26 axis. These findings, along with the role of *Fcgr3* in Amuc_1473‐induced regulation of osteoclast formation, uncover a dual mechanism by which Amuc_1473 suppresses osteoclast differentiation: transcriptional repression of *Fcgr3* via NELF‐E and translational upregulation of anti‐osteoclastic EGLN2 via RPL26.

### 2.6 Intermittent Fasting Alleviates Multifactorial Osteoporosis Partly by Regulating GM

**FIGURE 6:**
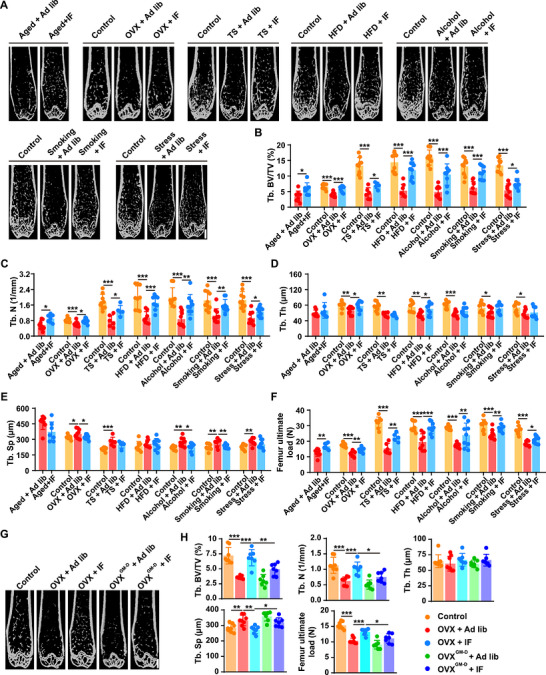
Intermittent fasting alleviates multifactorial osteoporosis partly by regulating gut microbiota. (A–E) µCT images (A) and quantification of Tb. BV/TV (B), Tb. N (C), Tb. Th (D), and Tb. Sp (E) in distal femurs from control mice and different detrimental factors‐induced osteoporotic mice subjected to ad libitum feeding (Ad lib) or intermittent fasting (IF). Scale bars: 1 mm. *n* = 5 (TS + IF), 6 (TS + Ad lib), 7 (Aged + IF), 8 (Aged + Ad lib, HFD + Ad lib , Stress + Ad lib), 10 (Control groups for OVX, Stress, and smoking models, Smoking + Ad lib), 9 (all other groups). (F) Three‐point bending measurement of femur ultimate load. *n* = 5–10 per group. (G) µCT images of distal femurs from Sham or OVX mice subjected to ad libitum feeding (control group and OVX + Ad lib group) or intermittent fasting (OVX + IF group), gut microbiota (GM)‐depleted OVX (OVX^GM‐D^) mice with ad libitum feeding (OVX^GM‐D^ + Ad lib), and OVX^GM‐D^ mice receiving intermittent fasting (OVX^GM‐D^ + IF). Scale bars: 1 mm. (H) Quantitative analysis of Tb. BV/TV, Tb. N, Tb. Th, and Tb. Sp. *n* = 7 (OVX + IF, OVX^GM‐D^ + Ad lib, OVX^GM‐D^ + IF) or 8 (Control, OVX + Ad lib). Data are mean ± SD. Statistics: unpaired, two‐tailed Student's *t*‐test (B–F; H: Control vs. OVX + Ad lib); two‐way ANOVA with Bonferroni post hoc test (H: comparisons among OVX groups). ^*^ *p* < 0.05, ^**^ *p* < 0.01, ^***^ *p* < 0.001.

We next explored whether intermittent fasting confers protection against osteoporosis induced by diverse deleterious stimuli, including natural aging, OVX‐induced estrogen deficiency, TS‐induced mechanical unloading, high‐fat diet, alcohol consumption, smoking, or psychological stress. We subjected mice exposed to different bone‐detrimental factors to either ad libitum feeding or four cycles of intermittent fasting regimens (each cycle consisting of alternate‐day fasting for one week followed by ad libitum feeding for the next week. µCT and three‐point bending tests demonstrated that intermittent fasting robustly improved trabecular bone mass and mechanical strength in aged mice, as well as in young mice exposed to the above bone‐compromising conditions, as revealed by significantly increased Tb. BV/TV, Tb. Th, Tb. N, or/and femur ultimate load, as well as markedly reduced Tb. Sp after intermittent fasting treatment (Figure 6A–F). These findings indicate that intermittent fasting exerts potent osteoprotective effects under multiple pathological contexts.

To determine whether the GM mediates these effects, we pre‐treated OVX mice with a broad‐spectrum antibiotic cocktail to deplete the intestinal microbiota. In this GM‐depleted OVX (OVX^GM‐D^) model, intermittent fasting retained significant, yet notably attenuated bone‐preserving effects compared to vehicle‐treated controls (Figure 6G,H). These results indicate that the anti‐osteoporotic effects of intermittent fasting are at least partially dependent on the modulation of GM.

### 2.7 Intermittent Fasting Enhances Gut *Akk* Abundance and Its Derivatives in the Bone

**FIGURE 7:**
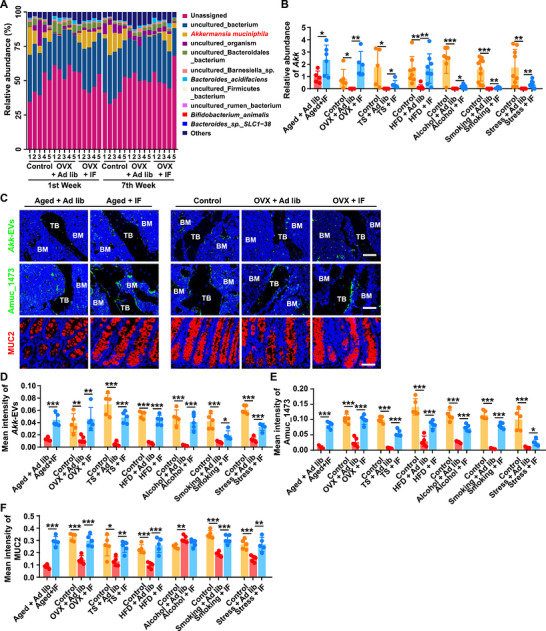
Intermittent fasting enhances gut *Akk* abundance and its derivatives in the bone. (A) 16S rRNA gene sequencing showing the relative abundance of the identified fecal microbiota at the species level. *n* = 3 per group. (B)  qRT‐PCR analysis of the relative abundance of gut *Akk* from different bone‐detrimental factors‐treated mice and control mice subjected to ad libitum feeding or intermittent fasting. *n* = 5 (Aged + Ad lib, OVX + Ad lib, OVX + IF, TS + Ad lib, Control group for TS model), 6 (Aged + IF, Control group for OVX model, TS + IF, Alcohol + Ad lib), 7 (Control group for Alcohol model, Stress + Ad lib), 8 (all groups for HFD model), 10 (Control group for smoking model), 9 (all other groups). (C–F) Representative staining images (C) and quantification of mean intensity of *Akk*‐EVs (D)‐ or Amuc_1473 (E)‐stained femur sections and mucin 2 (MUC2)‐stained intestinal tissues (F) from mice receiving the indicated treatments. Scale bar: 50 µm. *n* = 5 per group. Data are mean ± SD. Statistics: unpaired, two‐tailed Student's *t*‐test (B and D–F). ^*^ *p* < 0.05, ^**^ *p* < 0.01, ^***^ *p* < 0.001.

To determine whether intermittent fasting restores gut *Akk*, we profiled the GM of OVX mice subjected to ad libitum feeding or intermittent fasting using 16S rRNA gene sequencing. As shown in Figure 7A and Table S8, OVX markedly suppressed *Akk* abundance at the species level, while intermittent fasting robustly rescued its abundance, indicating a restorative effect on the gut microbial niche. We next evaluated the impact of intermittent fasting on *Akk* levels in the gut, as well as on *Akk*‐EVs and their signature protein Amuc_1473 in the bone, across multiple osteoporosis models. qRT‐PCR of fecal microbiota revealed that intermittent fasting significantly counteracted *Akk* depletion induced by aging, OVX, tail suspension, high‐fat diet, alcohol intake, cigarette smoke, and chronic stress (Figure 7B). Immunofluorescence staining demonstrated substantial reductions of both *Akk*‐EVs and Amuc_1473 in the bone under these pathological conditions, but their levels were consistently restored following intermittent fasting (Figure 7C–E). These results indicate that intermittent fasting broadly reinforces the gut‐bone axis by augmenting *Akk* abundance and facilitating the recovery of its functional mediators in bone.

Given that mucins are essential for the colonization and persistence of *Akk* in the gut niche [44], we explored whether intermittent fasting promotes mucin production. Immunofluorescence staining of small intestinal sections revealed that most of these bone‐detrimental factors profoundly suppressed mucin 2 (MUC2) expression, whereas intermittent fasting markedly restored MUC2 levels (Figure 7C,F), suggesting that intermittent fasting‐mediated enhancement of mucosal integrity may underlie the reestablishment of *Akk* and its downstream bone‐protective signals.

Previous studies have reported that the above‐described bone‐detrimental stimuli can induce hyperactivation or dysregulation of sympathetic nervous system (SNS) and/or hypothalamic‐pituitary‐adrenal (HPA) axis [45, 46, 47, 48, 49, 50, 51, 52, 53, 54, 55]. We then performed ELISA to measure the levels of norepinephrine (NE; the sympathetic neurotransmitter) and glucocorticoids (GC; the key effector of HPA axis) in intestinal tissues of mice subjected to different pro‐osteoporotic stressors. The results showed that all these factors induced statistically significant increases or trends toward elevation in intestinal NE and GC levels (Figure S8A). Moreover, direct treatment with NE or GC dose‐dependently suppressed *Akk* growth, with GC requiring lower concentrations than NE for inhibition (Figure S8B). These findings suggest that SNS hyperactivation and HPA axis‐mediated GC elevation represent key upstream mechanisms linking these stressors to intestinal *Akk* suppression.

**FIGURE 8:**
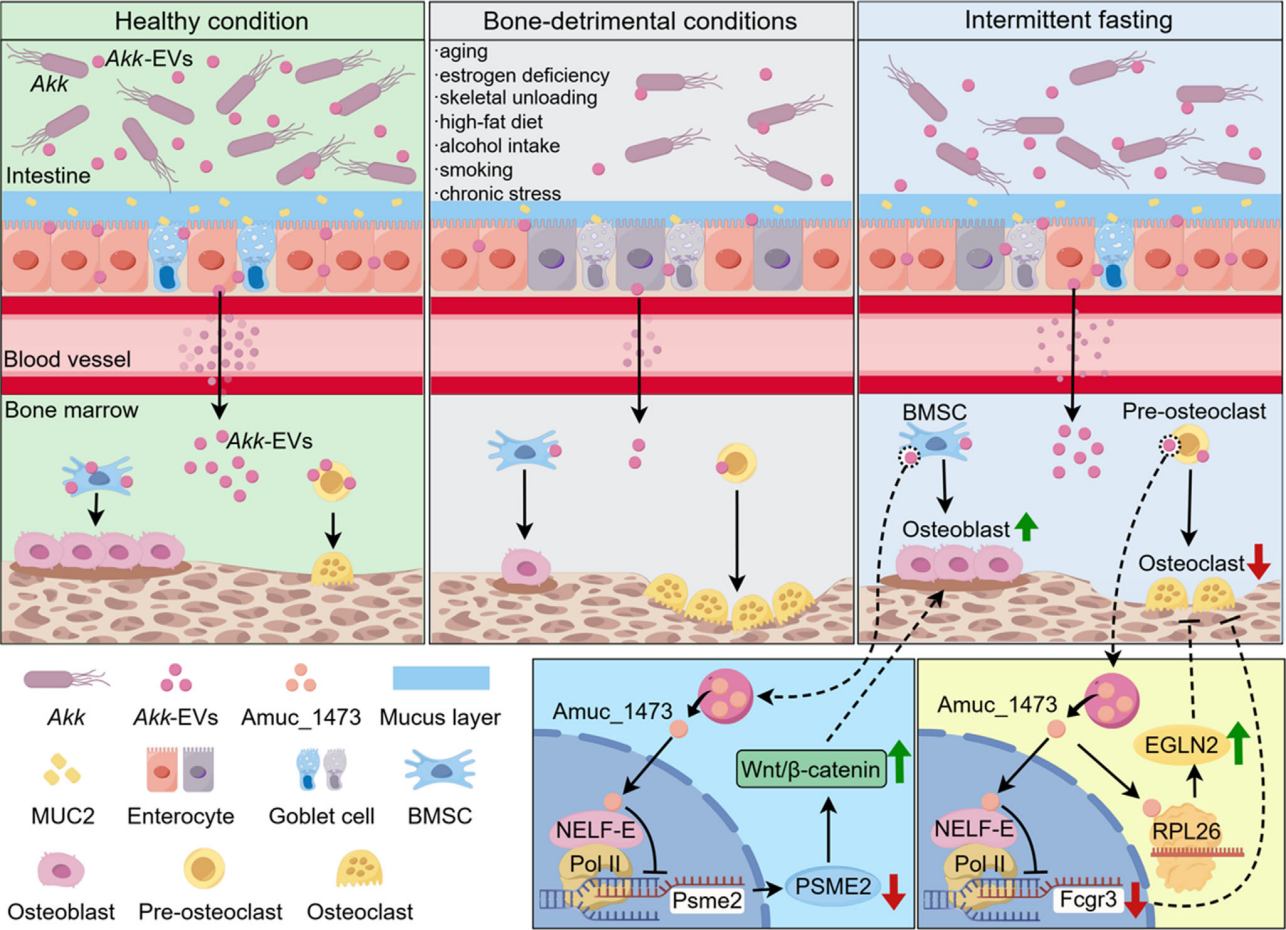
Schematic diagram showing the role of *Akk*‐EVs‐derived Amuc_1473 as a gut‐bone axis mediator in skeletal homeostasis and its restoration by intermittent fasting for osteoporosis therapy. This image was created using Figdraw, with the license number AOOTS30640.

Together, our study identifies Amuc_1473, a previously unknown protein enriched in *Akk*‐EVs, as a key mediator of gut‐bone crosstalk (Figure 8). Mechanistically, Amuc_1473 interacts with NELF‐E and RPL26 in bone cells to enhance osteogenesis and suppress osteoclastogenesis. Its levels decline under diverse pro‐osteoporotic conditions alongside neuroendocrine overactivation. Intermittent fasting restores Amuc_1473 levels partly by promoting intestinal mucin production and *Akk* colonization. Our findings establish Amuc_1473 as a gut bacteria‐derived systemic effector of bone homeostasis and reveal a microbiota‐targetable pathway for combating multifactorial osteoporosis.

## 3 Discussion

The concept of a gut–bone axis has gained increasing traction, yet the molecular messengers that transmit signals across this axis remain largely undefined. *Akk* is regarded as a next‐generation probiotic that inhabits the gut of both humans and animals, offering numerous beneficial biological functions [16, 18, 22, 56, 57]. However, the discovery of bioactive molecules responsible for the host‐protective effects of *Akk* is still in its early stages. It has been reported three key mechanisms—metabolite signaling, immune modulation, and gut‐bone axis crosstalk—through which *Akk* influences skeletal homeostasis [58]. Building on our prior evidence that *Akk*‐EVs can be delivered to bone tissue to alleviate osteoporosis [17], our study extends this line of work by identifying specific *Akk*‐EVs‐enriched proteins as important mediators of their bone‐protective effects. We demonstrate Amuc_1473, a previously uncharacterized protein secreted via *Akk*‐EVs, as a critical microbial effector capable of modulating bone remodeling at multiple levels. By unveiling a mechanism wherein Amuc_1473 binds transcriptional and translational regulators (NELF‐E and RPL26) to concurrently promote osteogenesis and inhibit osteoclastogenesis, we establish a direct, molecularly defined link between gut microbial activity and skeletal homeostasis.

In both prokaryotic and eukaryotic cells, EVs are key mediators of intercellular and inter‐organ crosstalk by transferring specific cargos to the recipient cells [11]. These vesicles protect their cargo molecules from the harsh conditions within the body, such as enzymatic degradation and acidic environments [59]. In our study, the identification of Amuc_1473 as a *Akk*‐EVs‐derived, systemically active protein with dual osteo‐anabolic and anti‐resorptive properties represents a conceptual advance in microbiome research. Amuc_1473 acts via well‐defined protein–protein interactions to regulate key transcriptional and translational nodes in bone cells. This mode of action opens up new opportunities for engineering gut‐derived proteins as next‐generation biologics tailored to modulate organ‐specific pathologies.

In addition, *Akk*‐EVs‐enriched proteins, particularly Amuc_1473, enhance gut barrier integrity, which may also contribute to their bone beneficial effects. This also suggests that that systemic delivery of *Akk*‑EV cargoes to bone via EVs occurs mainly through regulated translocation pathways rather than passive leakage across a compromised barrier. Bacterial EVs are known to cross the intestinal barrier via multiple, non‑exclusive routes—such as endocytosis, transcellular transport, and paracellular passage—allowing them to enter the circulation and reach distal organs [60]. Future work will be needed to define the precise molecular mechanisms by which *Akk*‐EVs traverse the intestinal barrier to accumulate in bone, and to identify the specific EV‐surface molecules that mediate their recognition and uptake by bone cells.

Strikingly, we found that Amuc_1473 abundance is diminished across a spectrum of osteoporotic stressors—including estrogen deficiency, aging, mechanical unloading, high‐fat diet, alcohol, smoking, and chronic stress—highlighting its broad relevance as a common molecular denominator of multifactorial bone loss. This suggests that distinct pathological stimuli may converge mechanistically by impairing gut‐derived protective signals, thereby compromising bone integrity. In this context, Amuc_1473 functions not only as a biomarker but also as an active inter‐organ communicator—capable of exerting endocrine‐like effects from the gut lumen to distant skeletal tissues.

Our findings further reveal an upstream neuroendocrine–intestinal axis during this process. SNS hyperactivation‐ and HPA axis dysregulation‐mediated NE and GC accumulation alongside intestinal MUC2 depletion are likely pivotal upstream regulators linking these stressors to gut *Akk* suppression and its derivative reduction. However, the precise regulatory circuitry linking neural signaling, intestinal mucus dynamics, *Akk*‐EV cargoes, and bone pathophysiology remains incompletely defined. Future in vitro/in vivo studies are warranted to decipher these neuro‐gut‐microbiome‐bone interactions at molecular resolution, potentially unveiling novel therapeutic axes for osteoporosis prevention and treatment.

Intermittent fasting diets have gained popularity in recent years due to its notable health benefits, without the need for daily calorie tracking or the restriction of specific foods [61]. Shaping a healthier GM contributes to various benefits of intermittent fasting [62]. Our study reveals that this non‐pharmacological dietary regimen markedly restores *Akk* and rescues bone deterioration induced by diverse pro‐osteoporotic factors. This effect likely involves the upregulation of MUC2 mucin biosynthesis, facilitating the expansion of *Akk* and enhancing *Akk*‐EVs‐mediated bacteria‐to‐host delivery of bioactive proteins such as Amuc_1473. These findings not only suggest intermittent fasting as a broadly applicable intervention for osteoporosis but also offer a mechanistic rationale linking diet, microbiota composition, mucosal biology, and bone metabolism.

## 4 Limitations of Study

While our study establishes Amuc_1473 as a key gut‐derived regulator of bone remodeling, several limitations remain and warrant future investigation. First, although multiple *Akk*‐EV‐enriched proteins exhibited bone‐protective effects, we prioritized Amuc_1473 due to its superior efficacy. The roles and targets of others (e.g., Amuc_1426, Amuc_1213) await elucidation to uncover potential synergies. Second, the upstream regulatory pathways by which diverse osteoporotic stimuli converge to impair mucin biosynthesis are not fully understood. Whether this process also involves SNS hyperactivation and HPA axis dysregulation warrants further investigation. Third, although intermittent fasting robustly restored gut MUC2 protein levels in most models, an exception was observed in alcohol‐induced bone loss model, where MUC2 levels were paradoxically elevated and unresponsive to intermittent fasting, suggesting mucin‐independent *Akk* regulation in specific contexts. Fourth, optimal dosing regimens (dose, frequency, and duration) for *Akk*‐EV‐derived proteins require dedicated pharmacodynamic and pharmacokinetic studies. Finally, many mechanistic gaps persist, including direct in vitro evidence of Amuc_1473–RPL26 binding and in vivo validation of NELF‐E/RPL26 cooperative roles in mediating Amuc_1473's anti‐osteoporotic effects; these warrant future investigation.

## 5 Method Details

### 5.1 Animal Experiments

Animal care and experimental procedures were approved by the Ethical Review Board at Xiangya Hospital of Central South University (NO. 202004111). All experimental mice were of the C57BL/6J strain and housed under specific pathogen‐free conditions. Different factors‐induced osteoporotic animal models were employed in this study.

(i) Senile osteoporosis mouse model: 3‐month‐old male mice were used as the young group, while 18‐month‐old male mice served as the natural aging group. The aged mice were employed as the senile osteoporosis model.
(ii) Postmenopausal osteoporosis mouse model: 3‐month‐old female mice were anesthetized and subjected to either bilateral OVX surgery (experimental model of postmenopausal osteoporosis) or sham operation as described previously [17, 45, 63].
(iii) Disuse‐induced osteoporosis mouse model: 3‐month‐old male mice were suspended by their tails using adhesive tape, ensuring complete unloading of the hindlimbs. The suspension lasted for 21 days, during which the animals were unable to bear weight on their hindlimbs, stimulating disuse‐induced bone loss.
(iv) High‐fat diet‐induced osteoporosis mouse model: 3‐month‐old male mice were fed a high‐fat diet (Research Diets D12492, providing 60% of kcal from fat) to induce obesity and metabolic dysfunction. The diet was replaced every 2–3 days to ensure freshness and prevent contamination. The intervention lasted for 8 weeks.
(v) Alcohol‐induced osteoporosis mouse model: 3‐month‐old male mice were orally administered daily with 300 µL of Chinese pure grain liquor (53% alcohol by volume) during the first week, followed by 200 µL every day during the second week. After an additional 2 weeks, the mice were sacrificed for sample collection.
(vi) Smoking‐induced osteoporosis mouse model: 3‐month‐old male mice received four cycles of cigarette smoke and fresh air: 30 min of cigarette smoke exposure in a chamber followed by a 5‐min interval in a well‐ventilated area. This treatment was conducted twice weekly for 8 weeks.
(vii) Chronic stress‐induced osteoporosis mouse model: 3‐month‐old male mice exposed to two or three stressors daily for four alternating cycles, each lasting 1 week, with a total duration of 4 weeks. The stressors included: moist bedding (200 mL water added into 100 g dry bedding) for 6 h, light‐dark cycles for four times (each time last for 30 min), forced swimming for 5 min in water at 30°C–32°C, cage tilted at 45°, physical restrain in 50 mL Eppendorf tube for 2 h, high‐platform tail suspension for 1 h, and bedding removal for 1.5 h. A schematic of the stressor administration was shown below.

**Table:** 

Time	9:00 am	12:00 pm	15:00 pm
Monday	Moist bedding		Light‐dark cycles
Tuesday	Forced swimming	Cage tilt	Physical restrain
Wednesday	Moist bedding		Light‐dark cycle
Thursday	Cage tilt	Physical restrain	Tail suspension
Friday	Light‐dark cycles	Forced swimming	Moist bedding
Saturday	Physical restrain		Cage tilt
Sunday	Forced swimming	No bedding	Light‐dark cycles

To assess the bone‐protective effects of intermittent fasting in the aforementioned osteoporotic models, these mice were subjected to an intermittent fasting regimen based on a 1:1 fasting schedule, as described in our recent study [64]. Under this regimen, mice were provided either ad libitum access to a standard chow diet (Hunan SJA Laboratory Animal Co., Ltd.; SJA‐slkjd003), except for the HFD model group, or subjected to various cycles of intermittent fasting. Each cycle consisted of one week of daily 24 h fasting followed by one week of ad libitum refeeding. During fasting days, animals had free access to water but no food; during refeeding, both water and food were available ad libitum. The regimen was conducted concurrently with other experimental procedures (e.g., tail suspension, chronic stress) throughout the intervention period. Feces, serum, small intestines, femurs, and tibias were collected to assess the levels of gut *Akk*, osteogenic or osteoclastic activity‐related indicators (OCN and CTX‐1), MUC2 expression, *Akk*‐EVs and their‐derived proteins in bone tissues, bone mass and strength, as well as osteoblast and osteoclast numbers, respectively.

To evaluate the preventive bone‐protective effects of *Akk*‐EV‐derived proteins, 3‐month‐old female mice (∼20–25 g body weight) underwent bilateral OVX or sham surgery under anesthesia. One week after surgery, OVX mice received intravenous injections of Amuc_1473, Amuc_1426, or Amuc_1231 at a dose of 4 µg per mouse in 100 µL PBS, equivalent to the maximal effective in vitro concentration (1 µg/mL) based on extracellular fluid volume estimates (∼20% body weight, or 4–5 mL/mouse). Control OVX and sham‐operated mice received 100 µL PBS vehicle. This dose aligns with prior *Akk* protein studies (3–10 µg/mouse) [65, 66, 67, 68, 69], balancing efficacy and safety. Mice had ad libitum access to water and standard chow (Hunan SJA Laboratory Animal Co., Ltd., SJA‐slkjd003). The treatment was conducted once a week for 8 weeks. Then, serum, femurs, tibias, and uteri were harvested for further analyses. Liver and kidney function‐related‐parameters were measured in serum samples using automated analyzers at Changsha Huancai Streaming Technology Co., Ltd.

### 5.2 *Akk* Culture and *Akk*‐EV Isolation

*Akk* (ATCC BAA‐835, Manassas, VA) was grown in Brain Heart Infusion (BHI) broth (BD Bioscience, San Jose, USA) with 0.5% porcine mucin (Sigma–Aldrich, St Louis, USA) and 0.05% L‐cysteine‐HCl (Sigma–Aldrich) as mentioned previously [17]. The culture supernatant was harvested and sequentially centrifuged at 2000 × g for 30 min and 10 000 × g for 30 min at 4°C to eliminate bacteria and larger particles. The supernatant was then passed through a 0.22‐µm filter (Millipore, Billerica, USA) and concentrated 100‐fold by centrifugation at 4000 × g in Amicon Ultra‐15 Centrifugal Filter Units (100 kDa; Millipore). *Akk*‐EVs were purified from the concentrated supernatant via OptiPrep density gradient centrifugation, as detailed in our prior work [11, 17]. The protein concentration in *Akk*‐EVs was measured using a commercial BCA kit (Thermo Fisher Scientific, Waltham, USA). The morphology and sizes of *Akk*‐EVs were detected by a Hitachi H‐7650 transmission electron microscope (Tokyo, Japan) and nanoparticle tracking analysis, respectively. *Akk*‐EVs were utilized immediately or preserved at −80°C for subsequent experiments.

To assess the effects of NE and GC on *Akk* growth, the bacterium (1 × 10^8^ CFU/mL) was cultured in medium supplemented with NE (MedChemExpress, New Jersey, USA) or GC (MedChemExpress) at 0, 1, 5, 10, 50, or 100 µm. 24 h later, bacterial growth was evaluated by measuring the optical density value at 600 nm (OD600).

### 5.3 Proteomic Analysis

Three biological replicates for *Akk*‐EVs and *Akk*, as well as BMSC or RAW264.7 treated with Amuc_1473‐His and anti‐His‐bound M2 agarose beads (Amuc_1473‐His group), or with the anti‐His‐conjugated beads only (control group), were prepared and sent to Jingjie PTM BioLab (Hangzhou, China) for 4D label‐free LC‐MS/MS proteomic analysis, with procedures detailed in our previous study [11]. MaxQuant search engine (v.1.6.15.0) or Proteome Discoverer (v2.4.1.15) were used to process the resulting MS/MS data. The false discovery rate (FDR) was maintained at <1% to ensure data reliability. Gene Ontology (GO) annotation and functional enrichment analyses were performed to characterize the identified proteins. The cut‐off values were set to |fold change| ≥ 2.0 or 1.5 and *p* < 0.05.

### 5.4 Recombinant Proteins, Antibodies, siRNAs, and Plasmids

Recombinant Amuc_1426‐His, Amuc_1473‐His, Amuc_1102‐His, Amuc_1631‐His, Amuc_0543‐His, and Amuc_1213‐His proteins were expressed in *E. coli* BL21 (DE3) or Rosetta 2 (DE3) stains by GenScript Biotech (Nanjing, China). The full‐length target gene was subcloned into a plasmid vector and transformed into *E.coli* for protein expression. After evaluating the expression and solubility by SDS‐PAGE and western blotting, the production system was scaled up, and the target proteins were purified. Protein purity was greater than 80% (Amuc_1426‐His: ≥ 85%; Amuc_1473‐His: ≥ 90%; Amuc_1102‐His: ≥ 90%; Amuc_1631‐His: ≥ 80%; Amuc_0543‐His: ≥ 90%; Amuc_1213: ≥ 85%). Endotoxin was < 0.07 EU/µg. Recombinant NELF‐E protein was purchased from (Proteintech, Chicago, USA). siRNAs were purchased from RiboBio Co., Ltd. (Guangzhou, China). Primary antibodies were obtained as follows: anti‐CD90, anti‐β‐catenin, anti‐NELF‐E, anti‐MUC2, anti‐POLDIP2, anti‐CARM1, anti‐OLFML1, anti‐GRB2, anti‐SMAD4, and anti‐EGLN2 (Proteintech); anti‐OCN (Servicebio, Wuhan, China); anti‐p‐β‐catenin (Cell Signaling Technology, Danvers, USA); anti‐RPL26 (Zenbio, Chengdu, China, or Abclonal, Wuhan, China); anti‐Sca‐1, anti‐CD29, anti‐CD44 (BioLegend, San Diego, USA); anti‐CD34 (Invitrogen, Carlsbad, USA); anti‐CD45 (BD Biosciences, San Jose, USA). NELF‐E‐Flag and RPL26‐Flag plasmids were packaged by GenScript Biotech. siRNAs targeting sequences were as follows: si*Bgn*: GGTCATATTTCTCCATCTT; si*Nelfe*: TGCAGAAGAAGTTCAACAA; si*Egln2*: GCATGCGGTACTATGGTAT; si*Psme2*: ACAACCATTTCCAAGTACT; si*Tmem119*: TGACCTTCCTCATCATGTT; si*Mvp*: GGACCAGTCAGAAGCTGAA; si*Fcgr3*: GTACGGAGAAATCTTCAAA.

### 5.5 Cell Culture

Primary BMSCs were isolated from the bones of C57BL/6J mice following procedures detailed in our previous study [63]. These cells were maintained in α‐Minimum Essential Medium (α‐MEM; Hyclone, Logan, USA) containing 15% fetal bovine serum (FBS; Gibco, Grand Island, USA) and 1% penicillin–streptomycin (PS; Cat. Solarbio, Beijing, China). RAW264.7 cells (ATCC; Rockville, USA) were cultured in high‐glucose Dulbecco's modified Eagle's medium (DMEM; Gibco) with 10% FBS and 1% PS. Cells were incubated at 37°C and 5% CO_2_. For cell characterization and functional assays, BMSCs at passages 2–3 and RAW264.7 at passages 5–7 were used.

Flow cytometric analysis was performed to assess Sca‐1, CD29, CD44, CD90, CD34, and CD45 expression on BMSCs. Multipotent differentiation potential was confirmed by inducing BMSCs toward osteogenic, chondrogenic, and adipogenic lineages in specialized medium (Cyagen Biosciences, Guangzhou, China), followed by ARS, Oil Red O (ORO), and Alcian Blue (AB) staining to detect calcium deposits, lipid droplets, and proteoglycan‐rich matrices on days 8, 16, and 28, respectively. TRAP staining was used to evaluate spontaneous differentiation in RAW264.7 cells using a commercial kit from Sigma‐Aldrich.

### 5.6 Osteogenic Differentiation Assay

To test the effects of *Akk*‐EVs‐enriched proteins on osteogenesis, BMSCs were seeded in 48‐well culture plates at a density of 1 × 10^5^ cells per well. These cells were cultured in α‐MEM containing 10% FBS and 1% PS until approximately 80% confluence. The medium was then replaced by osteogenic induction medium (Cyagen Biosciences) added with Amuc_1426, Amuc_1473, Amuc_1102, Amuc_1631, Amuc_0543, or Amuc_1213 at the dose of 1 µg/mL, or an equal volume of vehicle (solvent). The osteogenic medium was refreshed every other day. To investigate the downstream mediators of the pro‐osteogenic effects of Amuc_1473, BMSCs were transfected with siCon or siRNAs targeting *Bgn*, *Nelf‐e*, *Rpl26*, *Tmem119*, or *Psme2* prior to induction. After 48 h, the cells were incubated in osteogenic medium added with Amuc_1473 protein (1 µg /mL) or vehicle. Following 3 days of induction, total RNA was extracted for transcriptome sequencing. After differentiation for 8–10 days, the cells were stained with ARS solution (Cas9X, Suzhou, China) to assess mineralized nodule formation.

### 5.7 Osteoclastic Differentiation Assay

To assess the impact of *Akk*‐EVs‐enriched proteins on osteoclast formation, RAW264.7 cells were seeded in 48‐well plates (5 × 10^3^ cells per well) and cultured in high‐glucose DMEM containing 10% FBS and 1% PS. 24 h later, the medium was renewed with osteoclastic induction medium (high‐glucose DMEM + 10% FBS + 1% PS + 100 ng/mL RANKL) added with Amuc_1426, Amuc_1473, Amuc_1102, Amuc_1631, Amuc_0543, or Amuc_1213 at the dose of 1 µg/mL, or an equal volume of vehicle. The induction medium was renewed every two days. After 3 days of induction, total RNA was harvested for transcriptome sequencing. Following 8 days of differentiation, the cells were subjected to TRAP staining with a kit from Sigma–Aldrich. The number of osteoclasts (≥ three nuclei) in each well was quantified under an inverted microscope.

### 5.8 µCT Analysis

Femurs, tibias, or/and lumbar vertebrae (L3–L4) samples were immersion‐fixed in 4% paraformaldehyde (PFA) for 48 h and scanned using high‐resolution µCT (vivaCT80; SCANCO Medical AG, Bruettisellen, Switzerland) with settings of 11.4 µm pixel size, 45 kV, and 177 µA. Datasets were reconstructed using NRecon v1.6, analyzed with CTAn v1.9, and visualized via µCT Vol v2.0. The trabecular region of interest (ROI) of femurs and tibias spanned 0.6–1.2 mm proximal to the distal growth plate. ROI of L3–L4 encompassed the entire vertebral body. Measurements included Tb. BV/TV, Tb. Th, Tb. N, and Tb. Sp.

### 5.9 Biomechanical Test

The biomechanical properties of femurs were evaluated by three‐point bending test on an electromechanical testing system (Instron 3343; Instron, Canton, USA). Prior to testing, femur specimens were positioned on two supporting bars with an 8 mm span, with the patella facing upward. A conical indenter connected to the load cell applied a compressive force to the midpoint of the femur at a rate of 5 mm/min. The test continued until fracture occurred, and the maximum bending force was automatically recorded by the machine's software. The load‐displacement curve was generated to determine the ultimate load value (N).

### 5.10 Histological, Immunohistochemical, and Immunofluorescence Staining

Bone tissues were immersed in 4% PFA for 48 h, followed by decalcification in 10% EDTA (pH *7.4*) for one week. Colon tissues were fixed in 4% PFA for 48 h without decalcification. All specimens were dehydrated through a graded ethanol series, cleared in xylene, and embedded in paraffin. Paraffin‐embedded sections were cut into 5‐µm‐thick slices for TRAP staining using a kit from Servicebio, immunohistochemical staining for OCN with antibody from Servicebio, or immunofluorescence staining for MUC2 using antibody from Proteintech. Images were captured using an Olympus CX31 optical microscope (Olympus, Tokyo, Japan) or Zeiss ApoTome microscope (ZEISS, Oberkochen, Germany). OCN‐positive osteoblasts, TRAP‐positive osteoclasts, and MUC2‐stained area intensity were quantified. Osteoblast/osteoclast numbers were normalized to bone surface perimeter (N/mm), and MUC2 intensity to stained area.

For β‐catenin immunostaining, BMSCs from different treatment groups were fixed in 4% PFA for 15 min, permeabilized with 0.5% Triton X‐100 in PBS for 15 min, and blocked with goat serum for 1 h. Slides were incubated overnight at 4°C with primary anti‐β‐catenin (Proteintech), followed by secondary antibody (Abcam, Cambridge, UK) for 1 h at room temperature. Nuclei were counterstained with DAPI, and signals were detected using a Zeiss ApoTome microscope.

### 5.11 ELISA

The concentrations of OCN, PINP, and CTX‐1 in mouse or human serum were tested by commercial kits from Elabscience (Wuhan, China). The levels of LPS and Zonulin‐1 in mouse serum, along with NE and GC in mouse colon were measured with commercial kits from Hengyuan (Shanghai, China). For Amuc_1473 protein detection, custom ELISA kits were prepared using essential reagents from Biolegend (San Diego. USA) and Amuc_1473 primary antibody developed by GenScript Biotech. The levels of Amuc_1473 in human plasma EVs were assessed by these kits.

### 5.12 qRT‐PCR Analysis

Fresh fecal samples were collected in sterile tubes and immediately frozen at −80°C to minimize DNA degradation and microbial activity. Microbial DNA was extracted using the TIANamp stool DNA kit (Tiangen Biotech Co., Beijing, China). 16S rDNA gene of *Akk* was amplified and the abundance of *Akk* was detected by qRT‐PCR on FTC‐3000 real‐time PCR system (Funglyn Biotech Inc., Toronto, Canada). The relative abundance of *Akk* was calculated using the comparative Ct (2^−ΔΔCT^) method, with normalization to the bacterium universal 16S rRNA primers. To examine the effect of NELF‐E on the Amuc_1473‐induced suppression of *Psme2* and *Fcgr3* expression, BMSCs and RAW264.7 cells were transfected with siCon or si*Nelf‐e* before induction. 2 days later, these cells were treated with Amuc_1473 or vehicle under osteogenic and osteoclastic induction, respectively. 24 h later, the differentiated cells were harvested. Total RNA was extracted and reverse‐transcribed into cDNA using All‐in‐one first‐Strand cDNA Synthesis SuperMix (Novoprotein, Suzhou, China). qRT‐PCR was performed with *Gapdh* serving as the internal control. Primers were as follows: *m‐Egln2*: Forward, 5’‐CAGGCCCTGAATCAAGCTCTC‐3’, and Reverse 5’‐CTGGCAGTGGTCGTAGTA GC‐3’); *m‐Psme2*: Forward, 5’GAGAAGCCCGAAAACAGGTG‐3’, and Reverse, 5’‐AGAGCTGACTCAGGGATATGATT‐3’; *m‐Fcgr3*: Forward, 5’‐CAGAATGCACA CTCTGGAAGC‐3’, and Reverse, 5’‐GGGTCCCTTCGCACATCAG‐3’; *m‐Cldn1*: Forward, 5’‐GGGGACAACATCGTGACCG‐3’, and Reverse, 5’‐AGGAGTCGAAG ACTTTGCACT‐3’; *m‐Cldn2*: Forward, 5’‐CAACTGGTGGGCTACATCCTA‐3’, and Reverse, 5’‐CCCTTGGAAAAGCCAACCG‐3’; *m‐Cldn3*: Forward, 5’‐ACCAACTG CGTACAAGACGAG‐3’, and Reverse, 5’‐CAGAGCCGCCAACAGGAAA‐3’; *m‐Cldn15*: Forward, 5’‐ATGTCGGTAGCTGTGGAGAC‐3’, and Reverse, 5’‐GGACGG AAAGTCCCAGCAG‐3’; *m‐Ocln*: Forward, 5’‐TTGAAAGTCCACCTCCTTACAG A‐3’, and Reverse, 5’‐CCGGATAAAAAGAGTACGCTGG‐3’; *m‐Jam3*: Forward, 5’‐ CTGCGACTTCGACTGTACG‐3’, and Reverse, 5’‐TTCGGTTGCTGGATTTGAGA TT‐3’; *m‐Gapdh*: Forward, 5’‐AGGTCGGTGTGAACGGATTTG‐3’, and Reverse, 5’‐TGTAGACCATGTAGTTGAGGTCA‐3’; *m‐Akk*: Forward, 5’‐CCTTGCGGTTGGC TTCAGAT‐3’, and Reverse, 5’‐CAGCACGTGAAGGTGGGGAC‐3’; *m‐UniF340*: Forward, 5’‐ACTCCTACGGGAGGCAGCAGT‐3’, and Reverse, 5’‐ATTACCGCGG CTGCTGG C‐3’.

### 5.13 Preparation of Antibody Targeting *Akk*‐EVs and Amuc_1473

Serum containing specific antibodies targeting *Akk*‐EVs were prepared by FriendBio Technology (Wuhan, China) as described previously [17]. Briefly, New Zealand rabbits were immunized subcutaneously with *Akk*‐EVs after collecting pre‐immune serum and then received three booster injections at different time points. Nine days later, serum was collected for ELISA‐based antibody titer determination. Serum with the highest antibody titer was chosen for downstream experiments. Polyclonal antibodies against Amuc_1473 were generated by GenScript Biotech using a fast immunization protocol. Production details are considered proprietary and cannot be disclosed.

### 5.14 Transcriptome Sequencing

Total RNA was extracted from BMSCs or RAW264.7 treated with vehicle, *Akk*‐EVs, or Amuc_1473, and processed for transcriptome sequencing by Novogene Co., Ltd. (Beijing, China). Briefly, mRNA was purified using poly‐T oligo‐attached magnetic beads and reverse‐transcribed into cDNA with random hexamer primer and M‐MuLV Reverse Transcriptase. Second‐strand cDNA synthesis was conducted using DNA Polymerase I and RNase H. After adapter ligation and size selection (370‐420 bp), the library was amplified by PCR and purified using the AMPure XP system. Sequencing was performed on the Illumina NovaSeq platform. Raw reads were processed using fastp software to remove adapters, poly‐N sequences, and low‐quality reads, resulting in high‐quality clean data. These reads were mapped to the reference genome using Hisat2. Gene expression was quantified using featureCounts v1.5.0‐p3. FPKM values were calculated for normalization. Differential expression analysis was conducted with an adjusted *p* ≤ 0.05 and |log_2_(foldchange)| > 0.0 to determine statistical significance.

### 5.15 16s rRNA Gene Sequencing

Feces were collected from sham‐ or OVX‐operated mice subjected to ad libitum feeding or intermittent fasting. Microbial DNA was extracted and processed for 16s rRNA gene sequencing by Genesky Biotechnologies Inc. (Shanghai, China), following protocols detailed in our previous study [11]. The V3‐V4 variable regions of the bacterial 16S rRNA gene were amplified via PCR. The resulting amplicons were fragmented, adaptor‐ligated, and used to construct sequencing libraries. These libraries were sequenced on an Illumina MiSeq platform (Illumina, USA). Raw reads were processed using QIIME2, Mothur, and Usearch to remove unnecessary sequences (e.g., adaptor sequences, primer sequences, shorter or low‐quality sequences), filter errors, cluster sequences with high similarity (>97%) into operational taxonomic units (OTUs), and assign taxonomic identities based on reference databases. The abundance and diversity of microbial species were assessed to evaluate the composition and structure of GM.

### 5.16 Study Participants

Human blood samples were collected from healthy volunteers or patients aged 19‐ to 79‐year‐old. Patients had conditions such as mild‐to‐moderate diabetes or hypertension, osteoporosis, lumbar disc herniation, or disc degeneration. Inclusion criteria excluded individuals with life‐threatening chronic illnesses (e.g., severe cardiovascular disease, cancer), serious immunological/inflammatory/infectious diseases, or long‐term use of antibiotics or glucocorticoids. Plasma was isolated to assess osteogenic or osteoclastic activity‐related markers (OCN, PINP, and CTX‐1), and to harvest EVs using a kit from Yeasen (Shanghai, China). Amuc_1473 levels in plasma EVs were examined by ELISA. BMD was measured by dual‐energy X‐ray absorptiometry (DXA) at the Endocrinology Department of the Third Xiangya Hospital of Central South University. Associations between Amuc_1473 and osteogenic/osteoclastic markers, as well as Amuc_1473 and BMD were evaluated. This human observational study was approved by the Ethics Committee at the Third Xiangya Hospital of Central South University (No. 24085), with written informed consent obtained from all donors.

### 5.17 Statistics Analysis

Data are presented as mean ± SD or mean ± SEM. Comparisons between two groups was conducted using an unpaired two‐tailed Student's *t*‐test. For multiple group comparisons, one‐way ANOVA with Bonferroni post hoc correction was applied to assess intergroup differences. Pearson correlation analysis was performed to determine the correlation coefficient. Statistical significance was set at *p* < 0.05. All statistical analyses were performed using GraphPad Prism 9 (GraphPad Software, USA). The exact sample size (n) for each analysis was provided in the corresponding figure legend.

## Author Contributions

H.X., C.‐Y.C., and J.‐H.L. designed this study. C.‐Y.C., H.‐J.Z., J.‐H.L., H.X., S.‐S.R., and Z.W., wrote the manuscript. S.‐S.R., H.‐J.Z., Z.W., C.‐G.H., Y.‐J.T., Y.‐X.D., M.‐J.L., J.‐Y.L., Y.‐W.L., X.W., Y.L., T.‐F.W., Z.‐H.H., and Z.G. performed the experiments or/and analyzed the data. S.‐S.R., H.‐J.Z., C.‐Y.C., and Y.‐W.L. prepared the figures. Y.Z., Z.‐G.W., G.‐W.H., H.Y., X.‐Y.H., Z.‐X.W., S.‐Y.C., W.D., H.‐L.L., H.‐J.L., J.C., and P.C. provided technical support.

## Conflicts of Interest

H.X., Z.W., S.‐S.R., C.‐Y.C., Y.‐X.D., and Z.‐X.W. are inventors of a submitted patent application related to this article. All other authors declare no competing interests.

## Supporting information




**Supporting File 1**: advs75639‐sup‐0001‐TableS1.xlsx.

**Supporting File 2**: advs75639‐sup‐0002‐TableS2.xlsx.

**Supporting File 3**: advs75639‐sup‐0003‐TableS3.xlsx.

**Supporting File 4**: advs75639‐sup‐0004‐TableS4.xlsx.

**Supporting File 5**: advs75639‐sup‐0005‐TableS5.xlsx.

**Supporting File 6**: advs75639‐sup‐0006‐TableS6.xlsx.

**Supporting File 7**: advs75639‐sup‐0007‐TableS7.xls.

**Supporting File 8**: advs75639‐sup‐0008‐TableS8.xlsx.

**Supporting File 9**: advs75639‐sup‐0009‐TableS9.xlsx.

**Supporting File 10**: advs75639‐sup‐0010‐SuppMat.xlsx.

**Supporting File 11**: advs75639‐sup‐0001‐Figure_S1.pdf.

**Supporting File 12**: advs75639‐sup‐0002‐Figure_S2.pdf.

**Supporting File 13**: advs75639‐sup‐0003‐Figure_S3.pdf.

**Supporting File 14**: advs75639‐sup‐0004‐Figure_S4.pdf.

**Supporting File 15** advs75639‐sup‐0005‐Figure_S5.pdf.

**Supporting File 16** advs75639‐sup‐0006‐Figure_S6.pdf.

**Supporting File 17**: advs75639‐sup‐0007‐Figure_S7.pdf.

**Supporting File 18**: advs75639‐sup‐0008‐Figure_S8.pdf.

## Data Availability

Any information required to reanalyze the data reported in this paper is available from the corresponding authors upon request.
