# Supplementary material for: Amuc_1473 Links Gut Microbes to Skeletal Homeostasis and Counteracts Multifactorial Osteoporosis
Source: Adv Sci (Weinh). 2026 Jun 13;13(44):e23067. doi: 10.1002/advs.202523067 (PMC13335810; doi:10.1002/advs.202523067)
Supplement: Supplementary file 1 [file ADVS-13-e23067-s003.pdf]

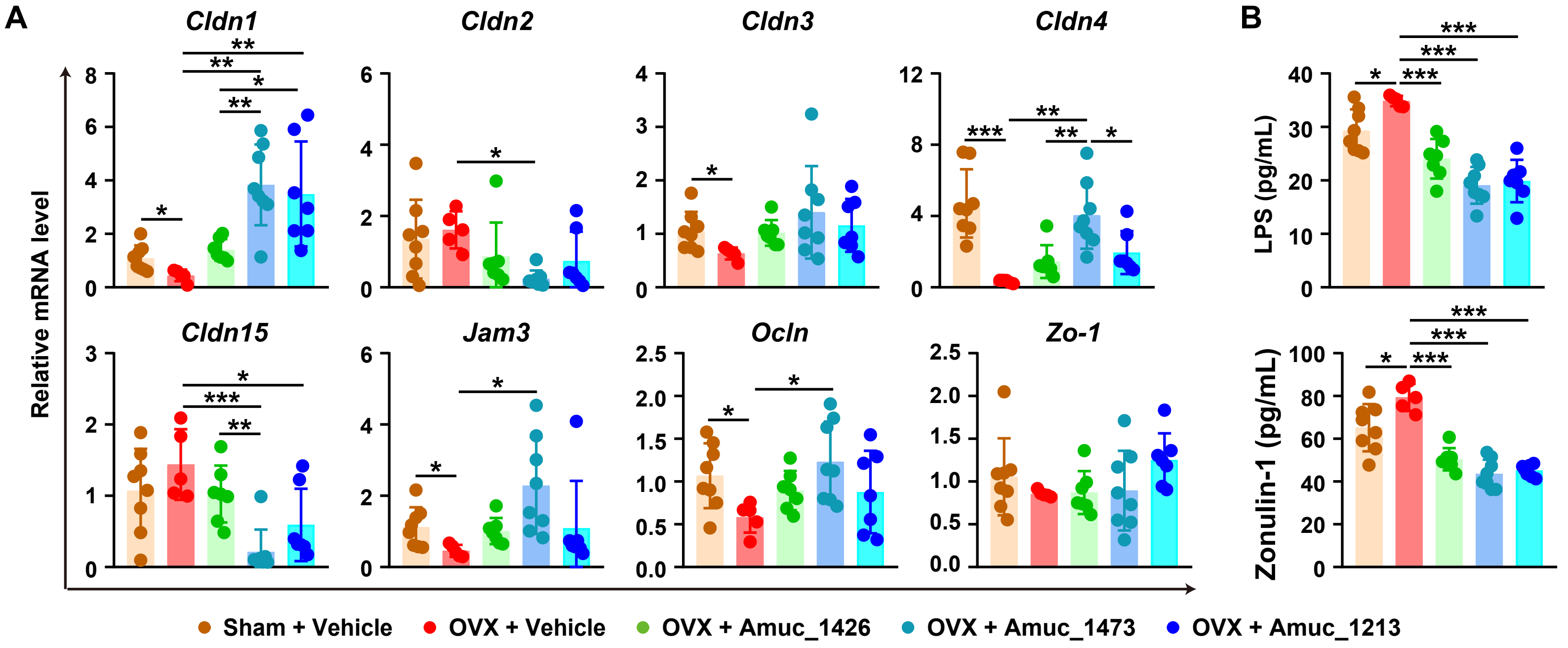
A
B
Cldn1
Cldn2
Cldn3
Cldn4
Cldn15
Jam3
Ocln
Zo-1
Relative mRNA level
LPS (pg/mL)
Zonulin-1 (pg/mL)
Sham + Vehicle
OVX + Vehicle
OVX + Amuc_1426
OVX + Amuc_1473
OVX + Amuc_1213
