# Supplementary material for: Amuc_1473 Links Gut Microbes to Skeletal Homeostasis and Counteracts Multifactorial Osteoporosis
Source: Adv Sci (Weinh). 2026 Jun 13;13(44):e23067. doi: 10.1002/advs.202523067 (PMC13335810; doi:10.1002/advs.202523067)
Supplement: Supplementary file 2 [file ADVS-13-e23067-s005.pdf]

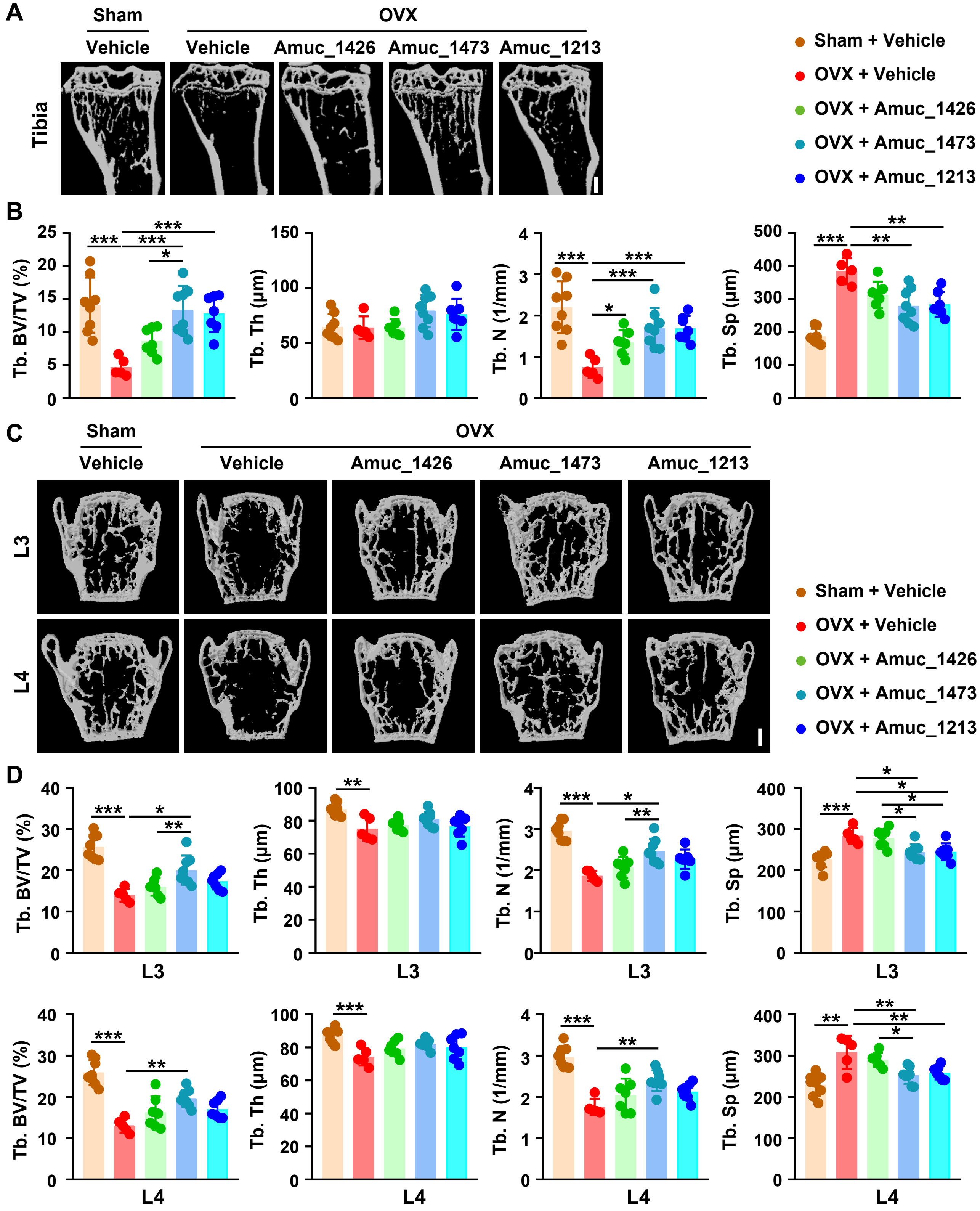

A
Sham
Vehicle
OVX
Vehicle
Amuc_1426
Amuc_1473
Amuc_1213
Tibia
Sham + Vehicle
OVX + Vehicle
OVX + Amuc_1426
OVX + Amuc_1473
OVX + Amuc_1213
B
Tb. BV/TV (%)
Tb. Th (μm)
Tb. N (1/mm)
Tb. Sp (μm)
C
L3
L4
D
L3
L4
