# Supplementary material for: Amuc_1473 Links Gut Microbes to Skeletal Homeostasis and Counteracts Multifactorial Osteoporosis
Source: Adv Sci (Weinh). 2026 Jun 13;13(44):e23067. doi: 10.1002/advs.202523067 (PMC13335810; doi:10.1002/advs.202523067)
Supplement: Supplementary file 3 [file ADVS-13-e23067-s007.pdf]

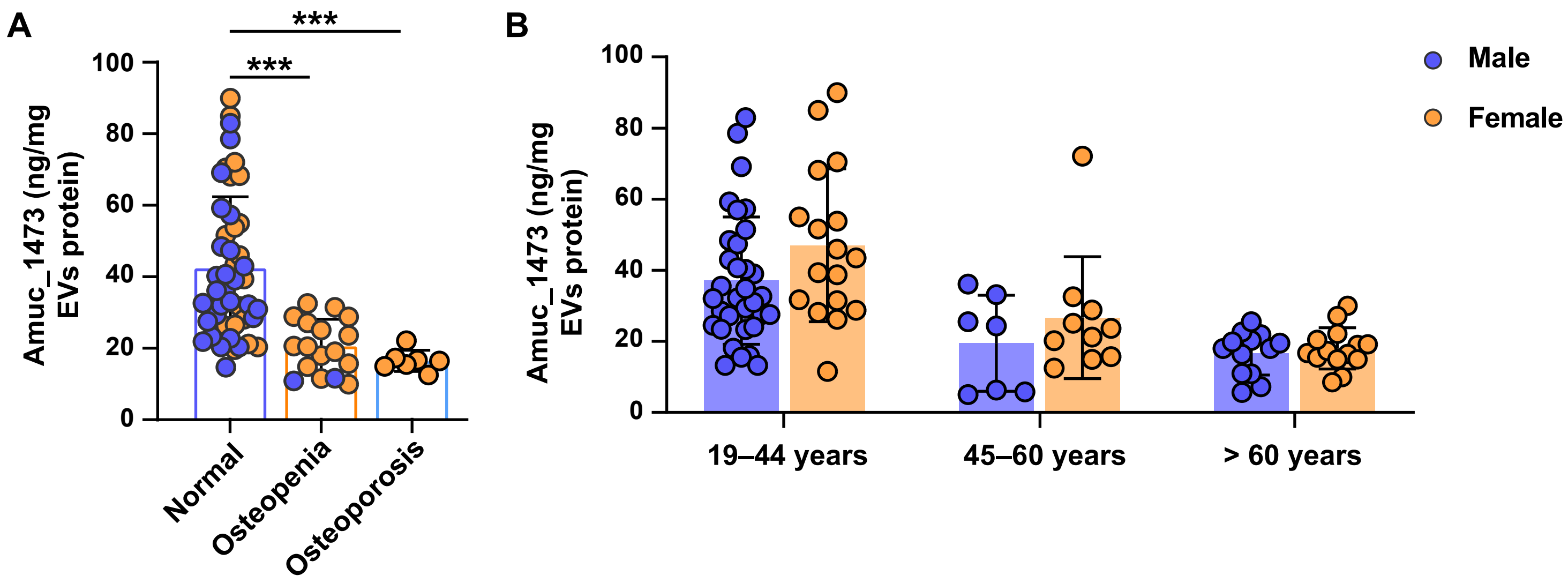
A
B
***
***
Amuc_1473 (ng/mg EVs protein)
100
80
60
40
20
0
Normal
Osteopenia
Osteoporosis
Male
Female
19–44 years
45–60 years
> 60 years
