# Supplementary material for: Amuc_1473 Links Gut Microbes to Skeletal Homeostasis and Counteracts Multifactorial Osteoporosis
Source: Adv Sci (Weinh). 2026 Jun 13;13(44):e23067. doi: 10.1002/advs.202523067 (PMC13335810; doi:10.1002/advs.202523067)
Supplement: Supplementary file 4 [file ADVS-13-e23067-s009.pdf]

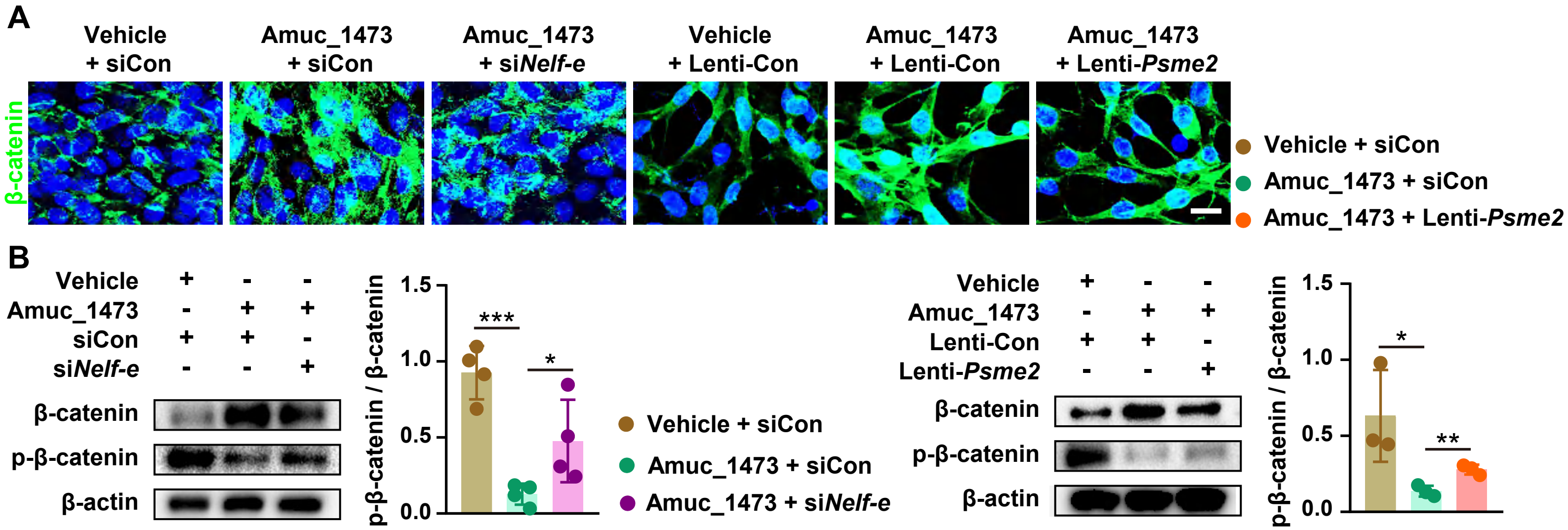
A
Vehicle + siCon
Amuc_1473 + siCon
Amuc_1473 + siNelf-e
Vehicle + Lenti-Con
Amuc_1473 + Lenti-Con
Amuc_1473 + Lenti-Psme2
β-catenin
Vehicle + siCon
Amuc_1473 + siCon
Amuc_1473 + Lenti-Psme2
B
Vehicle + - -
Amuc_1473 - + +
siCon + + -
siNelf-e - - +
β-catenin
p-β-catenin
β-actin
p-β-catenin / β-catenin
1.5
1.0
0.5
0.0
***
*
Vehicle + siCon
Amuc_1473 + siCon
Amuc_1473 + siNelf-e
Vehicle + - -
Amuc_1473 - + +
Lenti-Con + + -
Lenti-Psme2 - - +
β-catenin
p-β-catenin
β-actin
p-β-catenin / β-catenin
1.5
1.0
0.5
0.0
*
**
