# Supplementary material for: Amuc_1473 Links Gut Microbes to Skeletal Homeostasis and Counteracts Multifactorial Osteoporosis
Source: Adv Sci (Weinh). 2026 Jun 13;13(44):e23067. doi: 10.1002/advs.202523067 (PMC13335810; doi:10.1002/advs.202523067)
Supplement: Supplementary file 5 [file ADVS-13-e23067-s010.pdf]

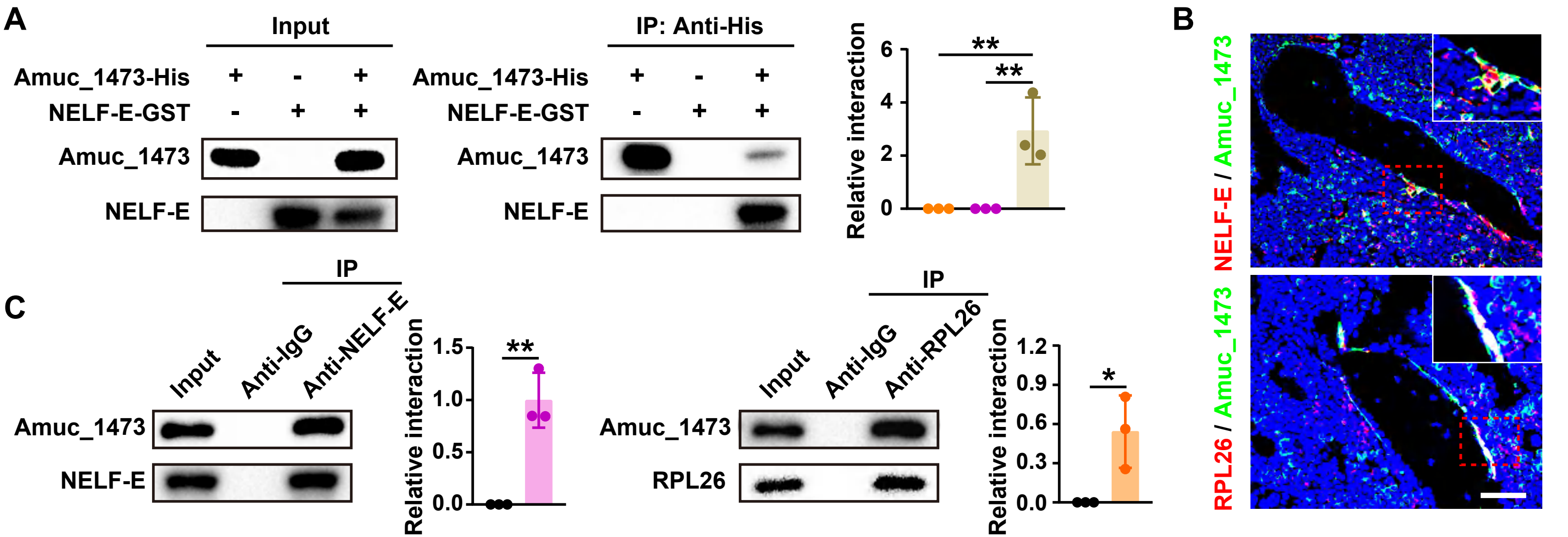

A
Input
IP: Anti-His
Amuc_1473-His
NELF-E-GST
Amuc_1473
NELF-E
Relative interaction
**
B
NELF-E / Amuc_1473
RPL26 / Amuc_1473
C
IP
Input
Anti-IgG
Anti-NELF-E
Anti-RPL26
Amuc_1473
NELF-E
RPL26
Relative interaction
*
