# Supplementary material for: Amuc_1473 Links Gut Microbes to Skeletal Homeostasis and Counteracts Multifactorial Osteoporosis
Source: Adv Sci (Weinh). 2026 Jun 13;13(44):e23067. doi: 10.1002/advs.202523067 (PMC13335810; doi:10.1002/advs.202523067)
Supplement: Supplementary file 6 [file ADVS-13-e23067-s014.pdf]

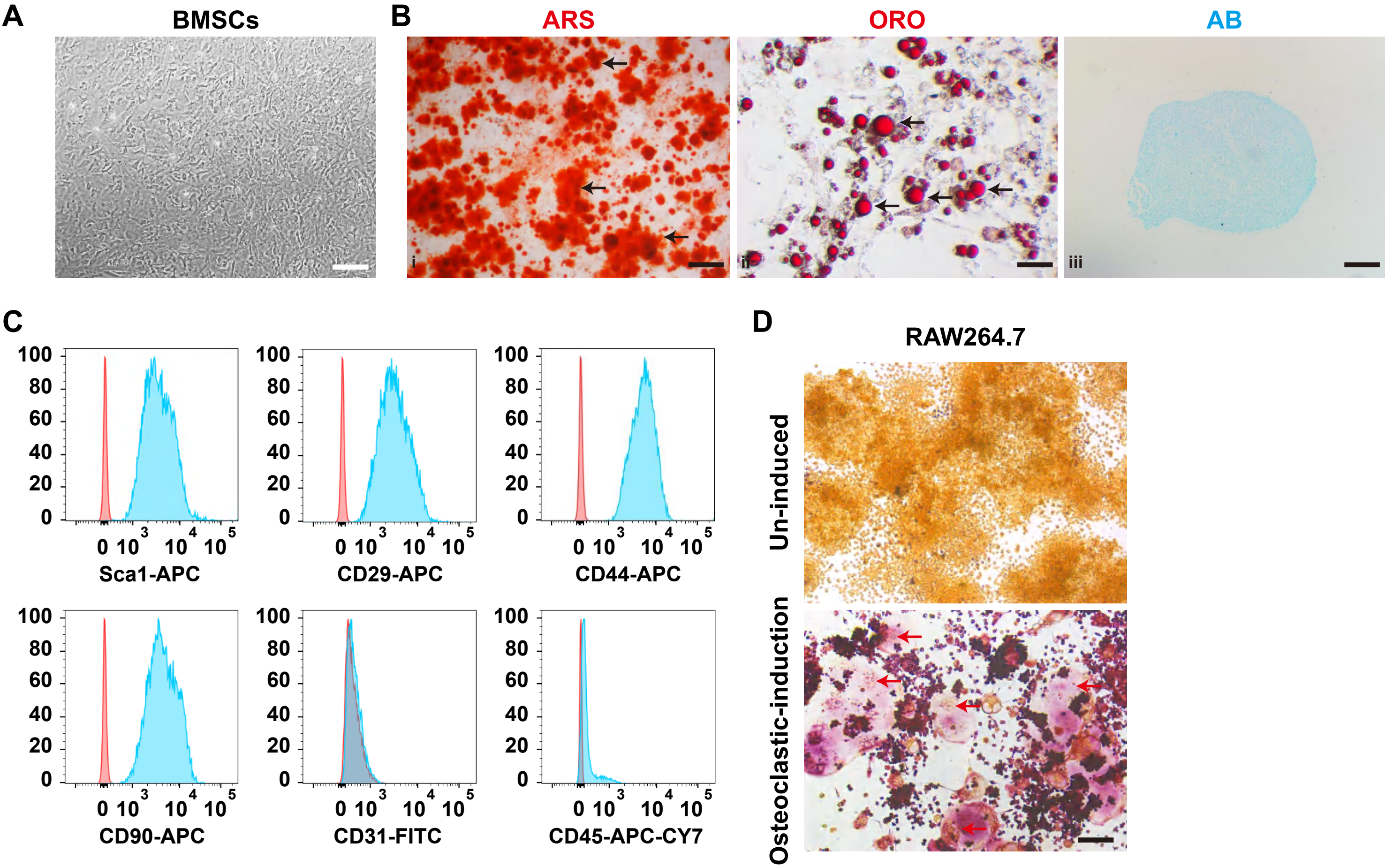
A
BMSCs
B
ARS
ORO
AB
i
ii
iii
C
Sca1-APC
CD29-APC
CD44-APC
CD90-APC
CD31-FITC
CD45-APC-CY7
D
RAW264.7
Un-induced
Osteoclastic-induction
