# Supplementary material for: Amuc_1473 Links Gut Microbes to Skeletal Homeostasis and Counteracts Multifactorial Osteoporosis
Source: Adv Sci (Weinh). 2026 Jun 13;13(44):e23067. doi: 10.1002/advs.202523067 (PMC13335810; doi:10.1002/advs.202523067)
Supplement: Supplementary file 7 [file ADVS-13-e23067-s016.pdf]

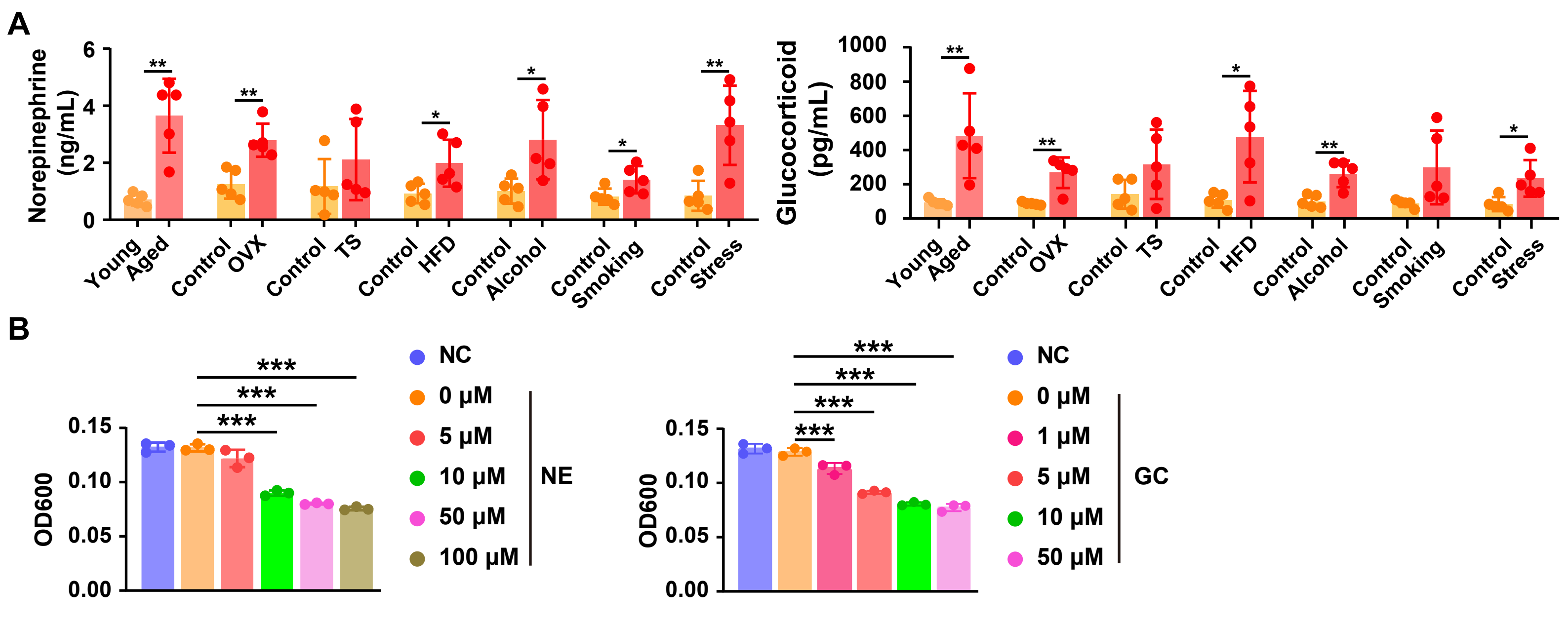
A
Norepinephrine (ng/mL)
0
2
4
6
**
**
*
*
*
**
Young
Aged
Control
OVX
Control
TS
Control
HFD
Control
Alcohol
Control
Smoking
Control
Stress
Glucocorticoid (pg/mL)
0
200
400
600
800
1000
**
**
*
**
*
B
OD600
0.00
0.05
0.10
0.15
***
***
***
NC
0 μM
5 μM
10 μM
50 μM
100 μM
NE
***
***
***
***
NC
0 μM
1 μM
5 μM
10 μM
50 μM
GC
