# Supplementary material for: Amuc_1473 Links Gut Microbes to Skeletal Homeostasis and Counteracts Multifactorial Osteoporosis
Source: Adv Sci (Weinh). 2026 Jun 13;13(44):e23067. doi: 10.1002/advs.202523067 (PMC13335810; doi:10.1002/advs.202523067)
Supplement: Supplementary file 8 [file ADVS-13-e23067-s018.pdf]

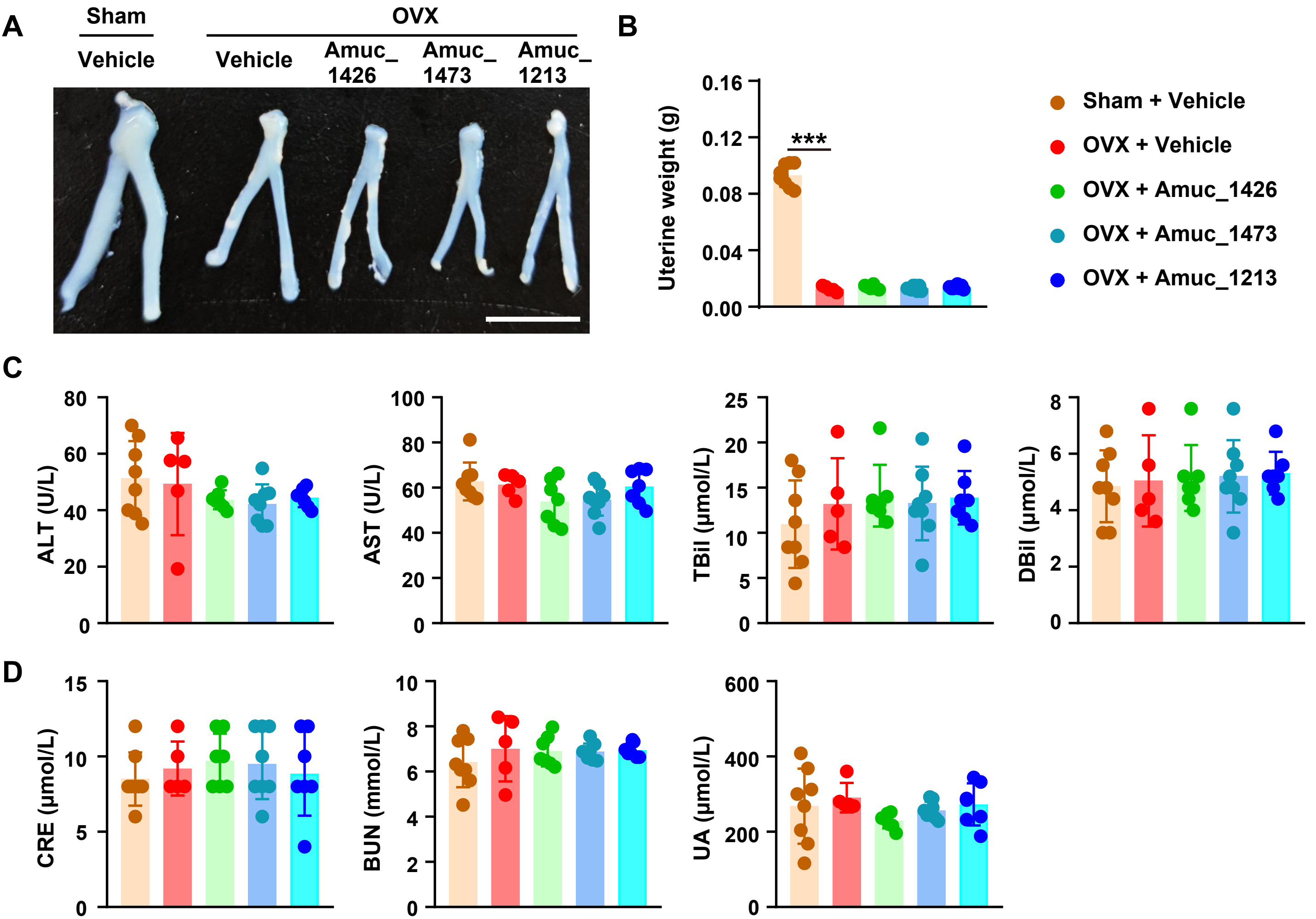

A
Sham
OVX
Vehicle
Vehicle
Amuc_ 1426
Amuc_ 1473
Amuc_ 1213
B
Uterine weight (g)
0.16
0.12
0.08
0.04
0.00
***
Sham + Vehicle
OVX + Vehicle
OVX + Amuc_1426
OVX + Amuc_1473
OVX + Amuc_1213
C
ALT (U/L)
AST (U/L)
TBil (μmol/L)
DBil (μmol/L)
D
CRE (μmol/L)
BUN (mmol/L)
UA (μmol/L)
