# Supplementary material for: Amuc_1473 Links Gut Microbes to Skeletal Homeostasis and Counteracts Multifactorial Osteoporosis
Source: Adv Sci (Weinh). 2026 Jun 13:e23067. Online ahead of print. doi: 10.1002/advs.202523067 (PMC13335810; doi:10.1002/advs.202523067)
Supplement: Supplementary file 10 — Supporting File 10: advs75639‐sup‐0010‐SuppMat.xlsx. [file ADVS-9999-e23067-s004.docx]

**Supplemental information**

**Amuc_1473 links gut microbes to skeletal homeostasis and counteracts multifactorial osteoporosis**

Shan-Shan Rao**^#^**, Hai-Jin Zeng^#^, Zun Wang, Chun-Gu Hong, Yi-Juan Tan, Yan-Xin Duan, Jing-Yao Luo, Ming-Jie Luo, Yi-Wei Liu, Xin Wang, Yi Luo, Teng-Fei Wan, Yong Zhou, Zheng-Guang Wang, Guo-Wen Hu, Hao Yin, Xin-Yue Hu, Zhen-Xing Wang, Ze-Hui He, Si-Yi Cheng, Wei Du, Zhe Guan, Hai-Li Lang, Hong-Ji Liu, Jia Cao, Peng Chen, Jiang-Hua Liu**^*^**, Hui Xie**^*^,** Chun-Yuan Chen**^*^**

^*^Corresponding authors:

Chun-Yuan Chen ([chency19@csu.edu.cn](mailto:chency19@csu.edu.cn)); Hui Xie ([huixie@csu.edu.cn](mailto:huixie@csu.edu.cn)); Jiang-Hua Liu (jianghua_liu@163.com)

**
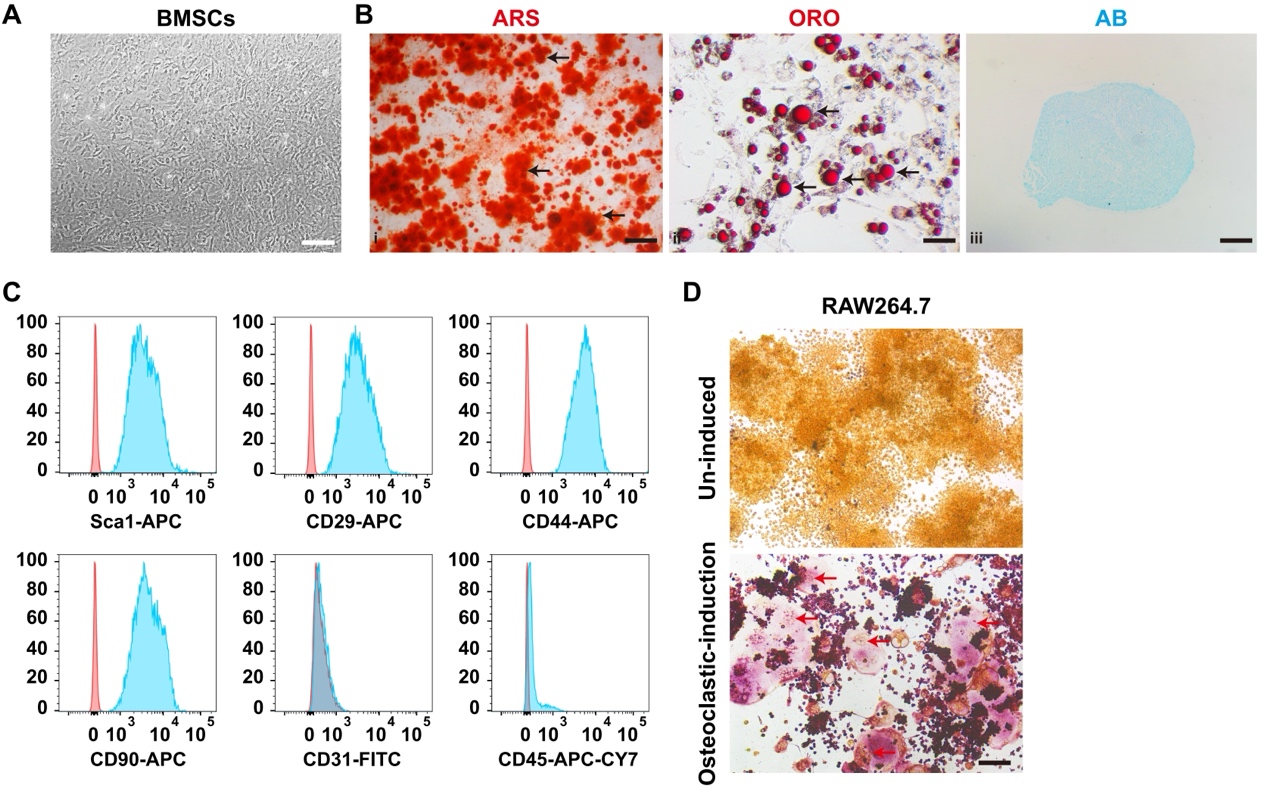
**

**Figure S1.** **Characterization of BMSCs and RAW264.7 cells.**

**(A)** Morphology of BMSCs visualized by optical microscopy. Scale bar: 100 μm. **(B)** Multi-lineage differentiation potential of BMSCs. Osteogenesis, adipogenesis, and chondrogenesis were confirmed by Alizarin Red S (ARS; mineralization areas showing red staining), Oil Red O (ORO; lipid droplets showing red staining), and Alcian Blue (AB; chondrocyte matrix showing blue staining), respectively. Scale bars: 200 μm (ARS), 100 μm (ORO), 500 μm (AB). **(C)** Flow cytometry histograms showing expression of BMSC‑positive (Sca-1, CD29, CD44, and CD90) and ‑negative (CD34 and CD45) surface markers. Isotype controls are shown in red. **(D)** Tartrate-resistant acid phosphatase (TRAP) staining of RAW264.7 cells under un-induced or osteoclastic induction. Scale bar: 200 μm.

**
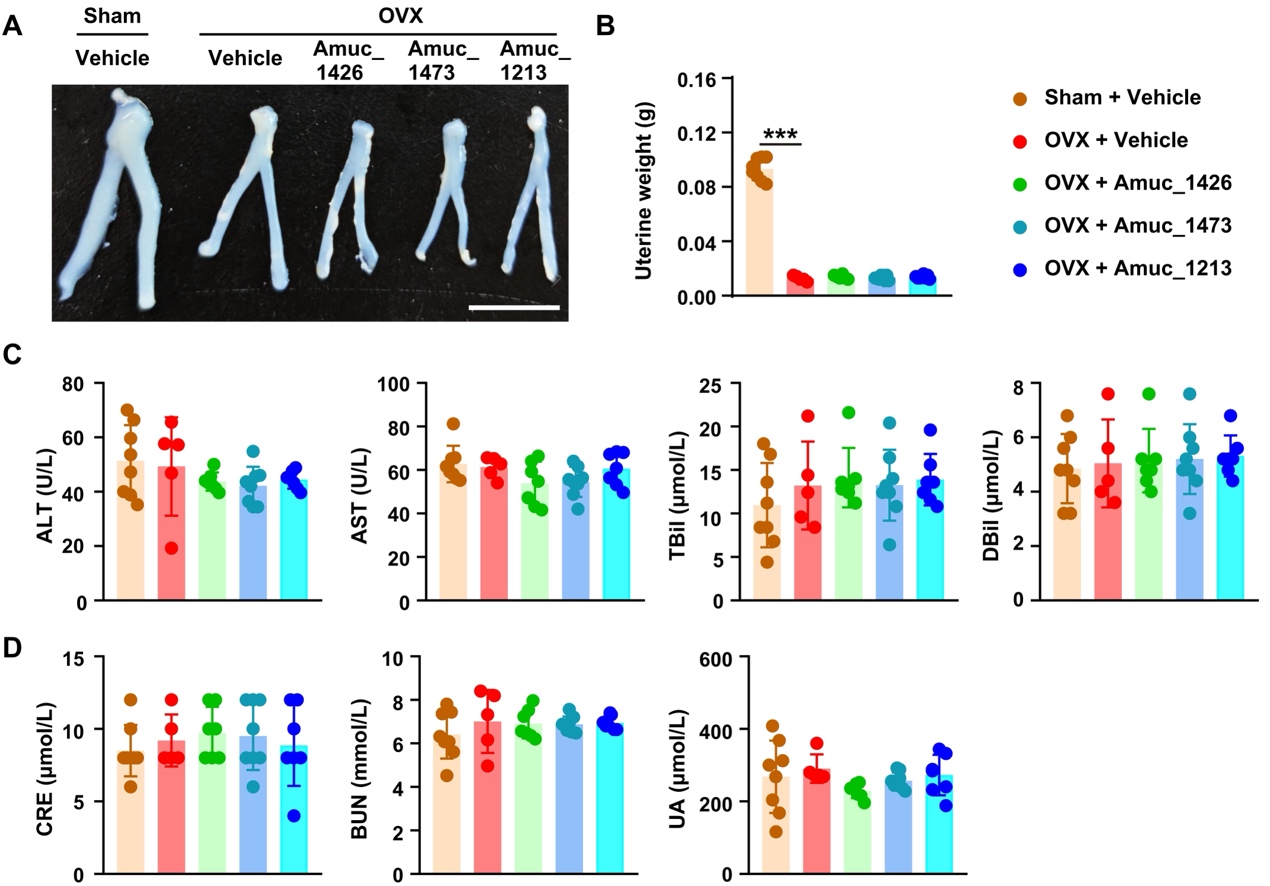
**

**Figure S2. Effects of *Akk*‑EV‑derived proteins on uterine size/weight and liver/kidney function in OVX mice.**

(**A–D**) Uterine images (**A**), uterus weights (**B**), and serum markers of liver and kidney function in sham- and OVX-operated mice receiving the indicated treatments. Scale bar, 1 mm. ALT, alanine transaminase; AST, aspartate transaminase; TBil, total bilirubin; DBil, direct bilirubin; CRE, creatinine; BUN, blood urea nitrogen; UA, uric acid. n = 5–8 per group. Data are mean ± SD. Statistics: unpaired, two-tailed Student's t-test (B**–**D: Sham + Vehicle versus OVX + Vehicle); one-way ANOVA with Bonferroni post hoc test (B**–**D: comparisons among OVX groups). ****P* < 0.001.

**
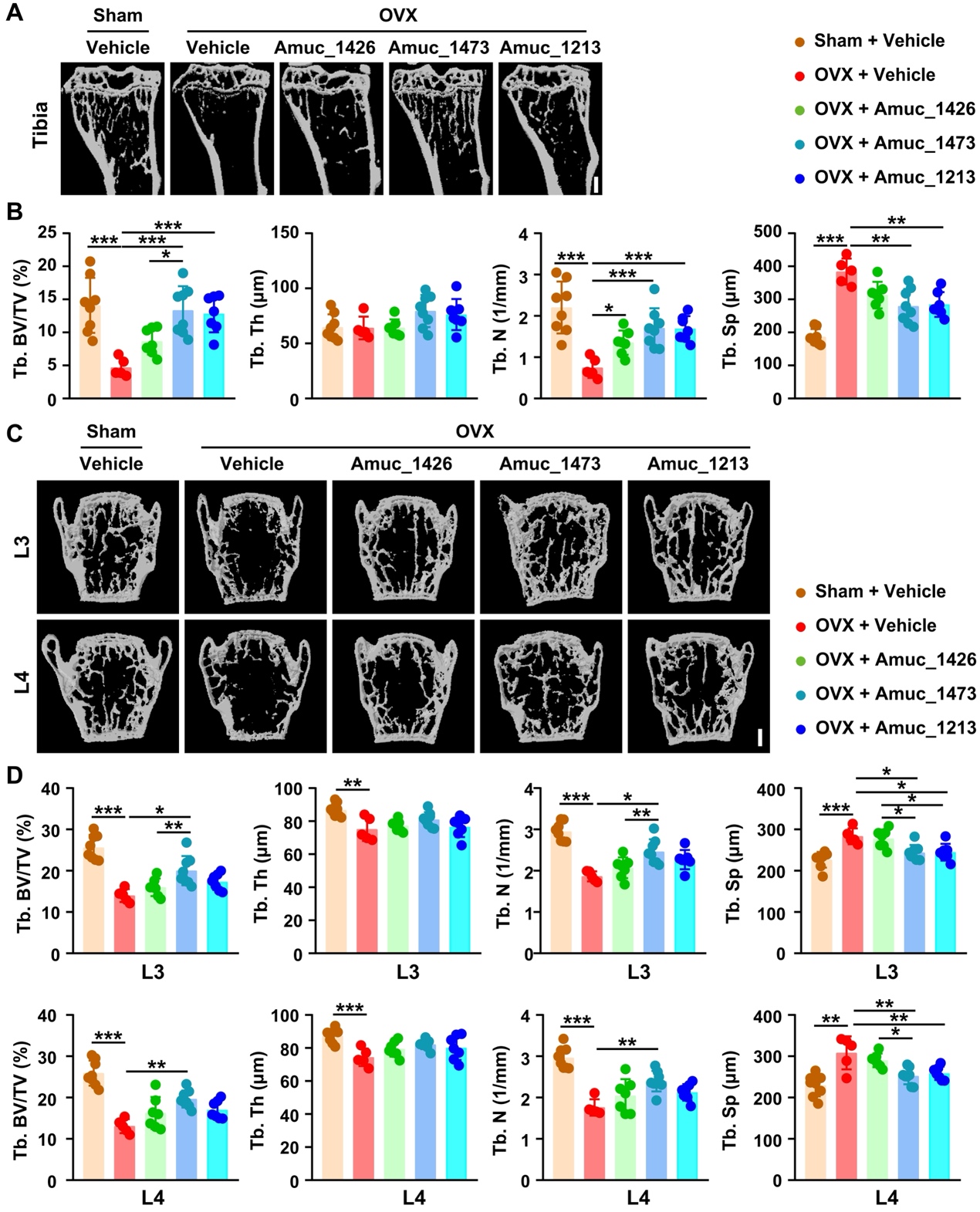
**

**Figure S3. Amuc_1473 robustly increases trabecular bone mass in tibia and lumbar vertebrae.**

**(A)** Representative µCT images of proximal tibia from sham- or OVX-operated mice receiving the indicated treatments. Scale bars: 1 mm. **(B)** Quantification of trabecular bone volume fraction (Tb. BV/TV), thickness (Tb. Th), number (Tb. N), and separation (Tb. Sp). *n* = 8 (Sham + Vehicle), 5 (OVX + Vehicle), 7 (OVX + Amuc_1426), 8 (OVX + Amuc_1473), 7 (OVX + Amuc_1213). **(C)** Representative µCT images of lumbar vertebrae (L3–L4) from sham- or OVX-operated mice receiving different treatments. Scale bars: 1 mm. **(D)** Quantification of Tb. BV/TV, Tb. Th, Tb. N, and Tb. Sp. *n* = 5-8 per group. Data are mean ± SD. Statistics: unpaired, two-tailed Student's t-test (B**,** D: Sham + Vehicle versus OVX + Vehicle); one-way ANOVA with Bonferroni post hoc test (B**,** D: comparisons among OVX groups). **P* < 0.05, ***P* < 0.01, ****P* < 0.001.

**
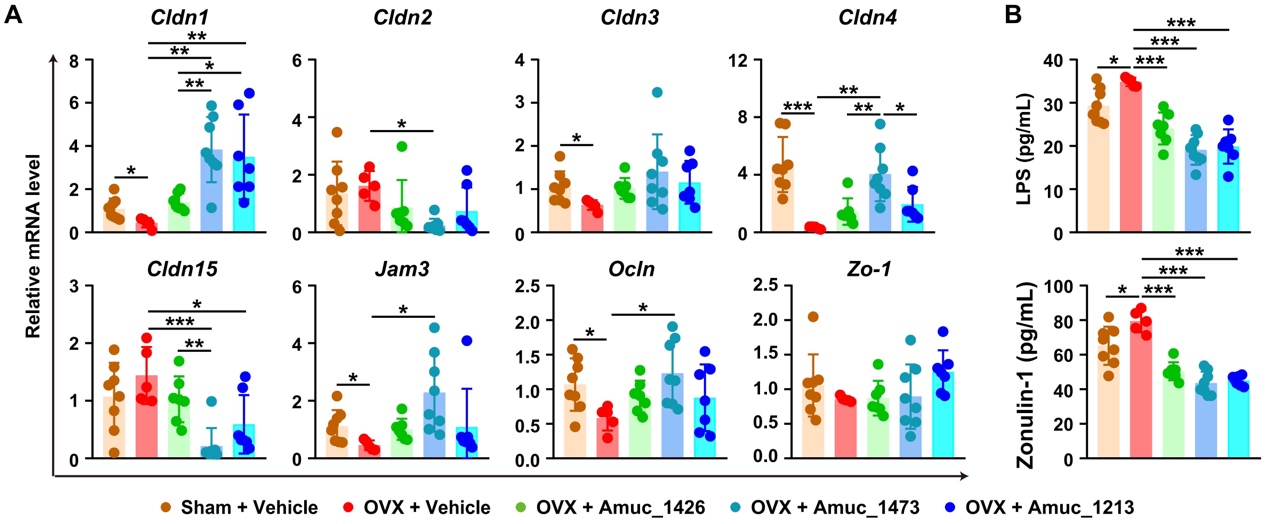
**

**Figure S4. Amuc_1473 potently restores gut barrier integrity in OVX mice.**

**(A)** qRT-PCR analysis of intestinal barrier-related gene expression in sham- or OVX-operated mice receiving the indicated treatments. Genes include *Claudin‑1* (*Cldn1*), *Cldn2*, *Cldn3*, *Cldn4*, *Cldn15*, junctional adhesion molecule 3 (*Jam3*), *Occludin* (*Ocln*), and Zonula Occludens‑1 (*Zo-1*). *n* = 8 (Sham + Vehicle), 5 (OVX + Vehicle), 7 (OVX + Amuc_1426), 8 (OVX + Amuc_1473), 7 (OVX + Amuc_1213). **(B)** Serum ELISA for lipopolysaccharide (LPS) and zonulin-1 levels. *n* = 5-8 per group. Data are mean ± SD. Statistics: unpaired, two-tailed Student's t-test (A**,** B: Sham + Vehicle versus OVX + Vehicle); one-way ANOVA with Bonferroni post hoc test (A**,** B: comparisons among OVX groups). **P* < 0.05, ***P* < 0.01, ****P* < 0.001.

**
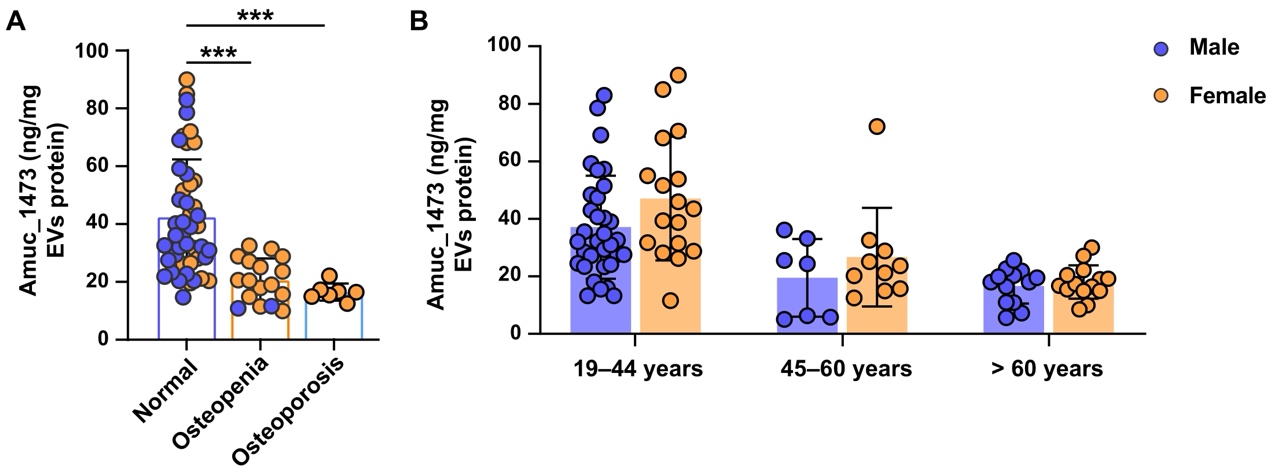
**

**Figure S5. Amuc_1473 declines in humans with osteopenia and osteoporosis.**

**(A)**Amuc_1473 levels in plasma EVs from humans with normal bone mass (n = 28 male/19 female), osteopenia (n = 2 male/15 female), or osteoporosis (n = 0 male/7 female). **(B)** Amuc_1473 levels in plasma EVs stratified by sex and age group (19–44 years: n = 31 male/17 female; 45–60 years: n = 7 male/10 female; >60 years: n = 13 male/14 female). Data are mean ± SD. Statistics: one-way ANOVA with Bonferroni post hoc test (A); unpaired, two-tailed Student's t-test (B). ****P* < 0.001.

**
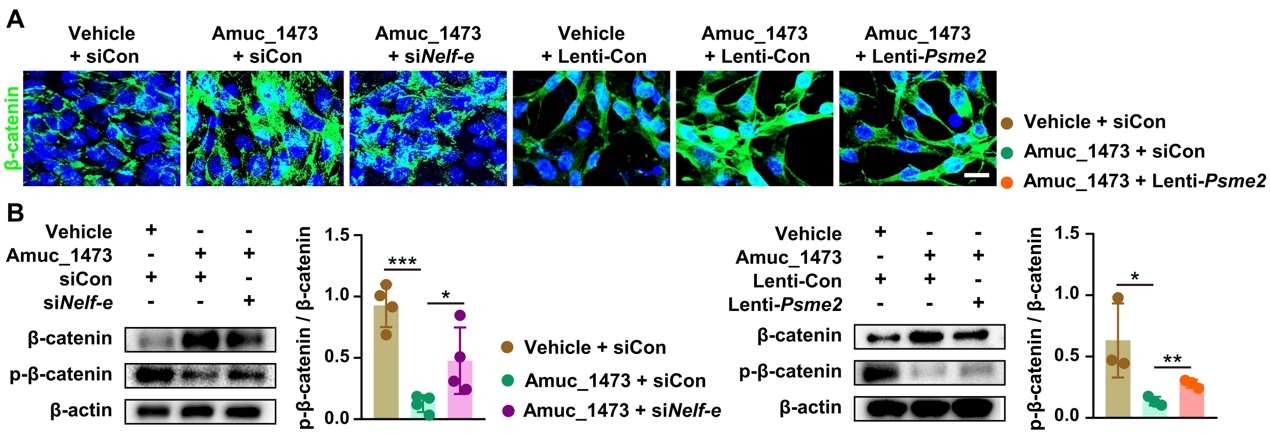
**

**Figure S6. Amuc_1473 activates Wnt/β‑catenin signaling via NELF-E and PSME2.**

**(A)** Immunofluorescence staining of β‑catenin in BMSCs after different treatments. Scale bar: 50 μm. **(B)** Western blot analysis of phosphorylated and total β-catenin protein in BMSCs following different treatments. Data are mean ± SEM. Statistics: unpaired, two-tailed Student's t-test (B). **P* < 0.05, ***P* < 0.01, ****P* < 0.001.

**
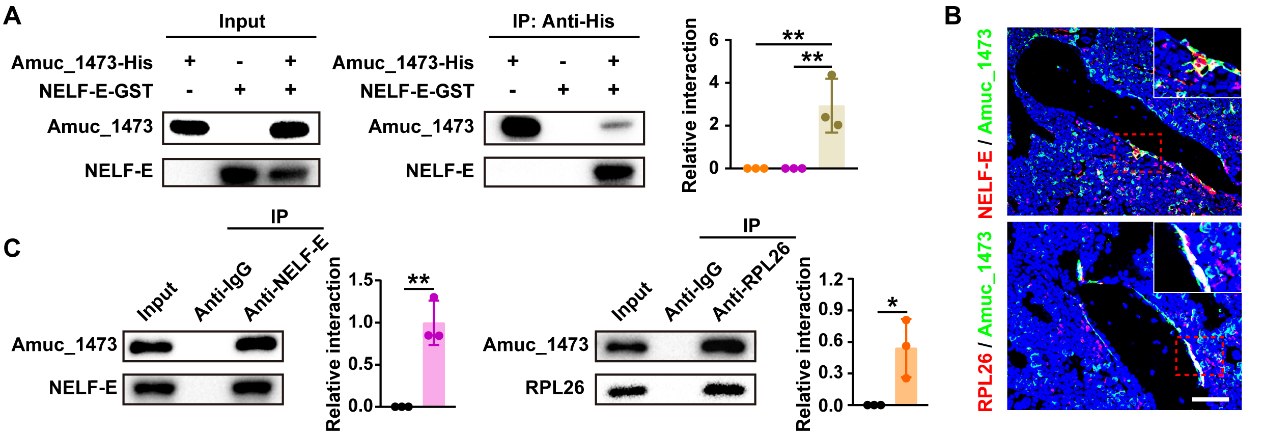
**

**Figure S7. Amuc_1473 interacts with NELF-E and RPL26 *in vitro* or/and *in vivo*.**

**(A)** *In vitro* binding between purified recombinant Amuc_1473-His and NELF-E-GST proteins assessed by anti-His pull-down assay. **(B)** Immunofluorescence co‑localization of Amuc_1473 with NELF‑E/RPL26 in femurs from 5-month-old mice. Scale bar: 20 μm. **(C)** *In vivo* co-immunoprecipitation of Amuc_1473 with NELF‑E/RPL26 using anti‑NELF‑E- or anti-RPL26 antibodies in bone lysates of 5-month-old mice. Statistics: one-way ANOVA with Bonferroni post hoc test (A); unpaired, two-tailed Student's t-test (C). Data are mean ± SEM. **P* < 0.05, ***P* < 0.01.

**
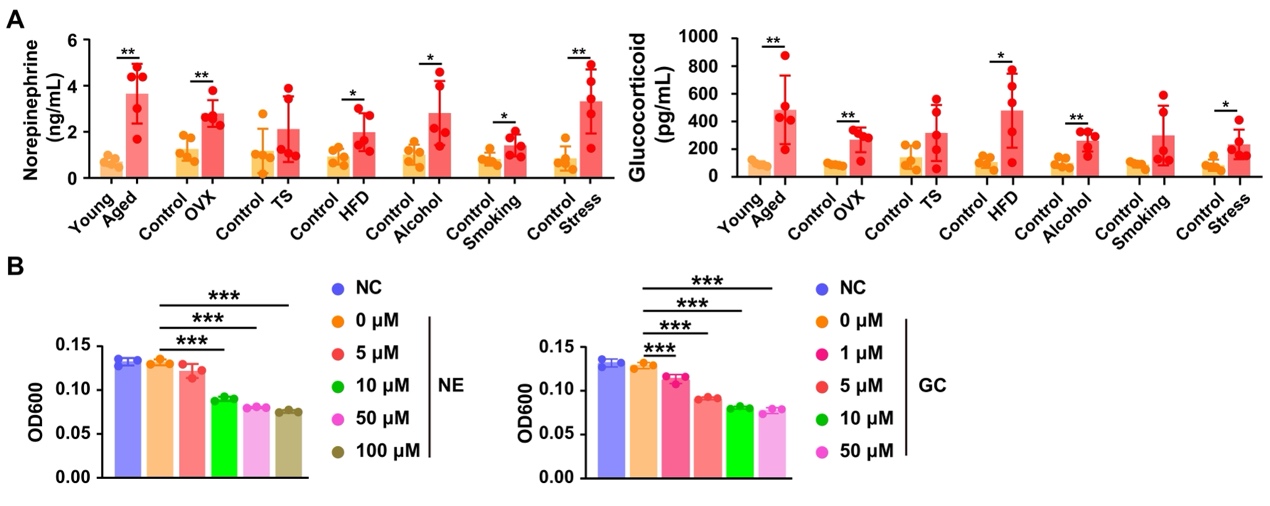
**

**Figure S8. Norepinephrine and glucocorticoids elevated by osteoporotic stressors suppress *Akk* growth.**

**(A)** Serum ELISA for norepinephrine (NE) and glucocorticoids (GC) in mice subjected to the indicated pro-osteoporotic stressors. *n =* 5 per group. **(B)** *In vitro* growth of *Akk* cultured with increasing concentrations of NE or GC, measured by optical density at 600 nm (OD600). *n =* 3 per group. Data are mean ± SD. Statistics: unpaired, two-tailed Student's t-test (A); one-way ANOVA with Bonferroni post hoc test (B). **P* < 0.05, ***P* < 0.01, ****P* < 0.001.

**Table legends**

**Table S1**: Differentially expressed proteins between *Akkermansia muciniphila* (*Akk*) and its derived extracellular vesicles (*Ak*k-EVs) by proteomic analysis.

**Table S2**: Functional classification of proteins highly enriched in *Akk*-EVs relative to *Akk* by gene ontology analysis.

**Table S3**: Proteins differentially pulled down by Amuc_1473-His protein compared to controls in BMSCs under osteogenic condition.

**Table S4**: Differentially expressed genes in response to *Akk*-EVs or Amuc_1473 treatment in BMSCs under osteogenic condition by transcriptome sequencing.

**Table S5**: Proteins differentially pulled down by Amuc_1473-His protein compared to controls in RAW264.7 cells under osteoclastic induction.

**Table S6**: Differentially expressed genes in response to *Akk*-EVs or Amuc_1473 treatment in RAW264.7 cells under osteoclastic induction.

**Table S7**: Predicted mRNAs with high affinity for RPL26 by catRAPID omics v2.1.

**Table S8**: 16S rRNA gene sequencing of fecal microbiota in mice receiving different treatments.

**Table S9**: Full names of abbreviations used in this study.
