# Supplementary figures and images for: Amuc_1473 Links Gut Microbes to Skeletal Homeostasis and Counteracts Multifactorial Osteoporosis
Source: Adv Sci (Weinh). 2026 Jun 13:e23067. Online ahead of print. doi: 10.1002/advs.202523067 (PMC13335810; doi:10.1002/advs.202523067)

**A****BMSCs**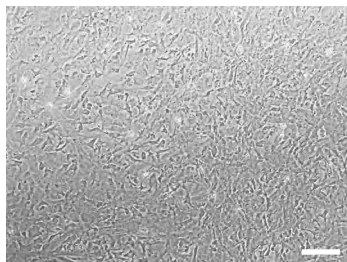**B****ARS**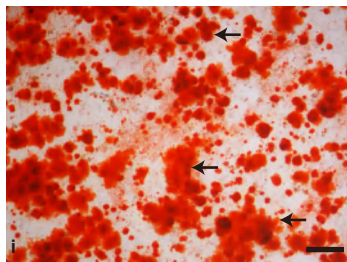**ORO**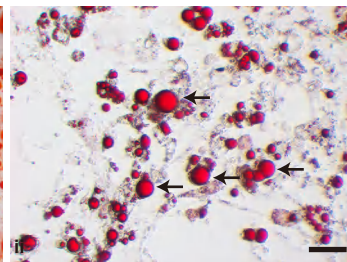**AB**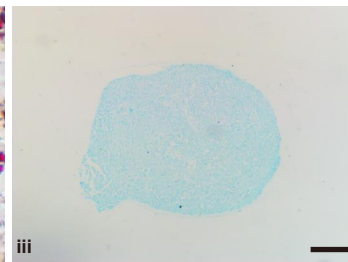**C**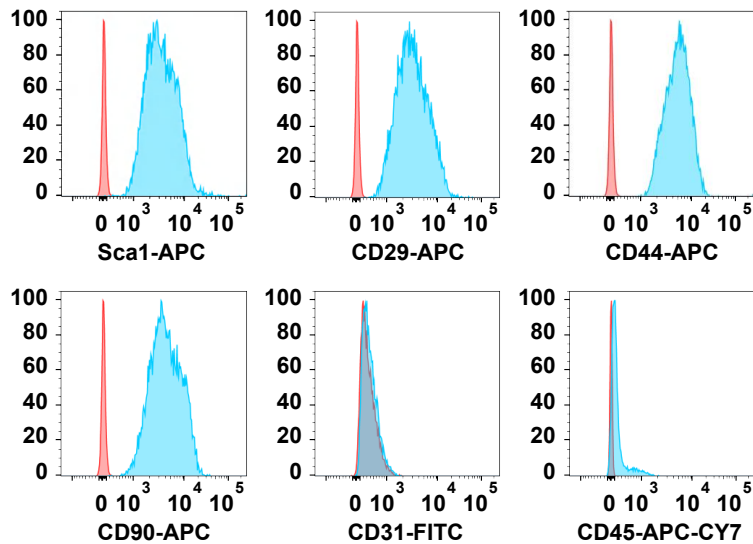**D****RAW264.7****Un-induced**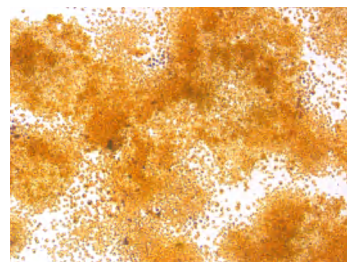**Osteoclastic-induction**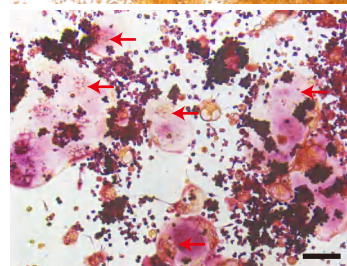

Supplement: Supplementary file 11 — Supporting File 11: advs75639‐sup‐0001‐Figure_S1.pdf. [file ADVS-9999-e23067-s012.pdf]

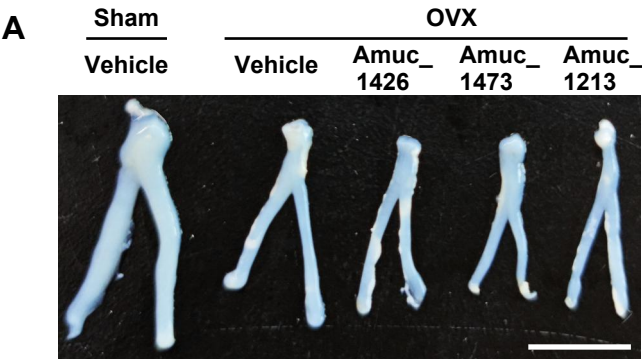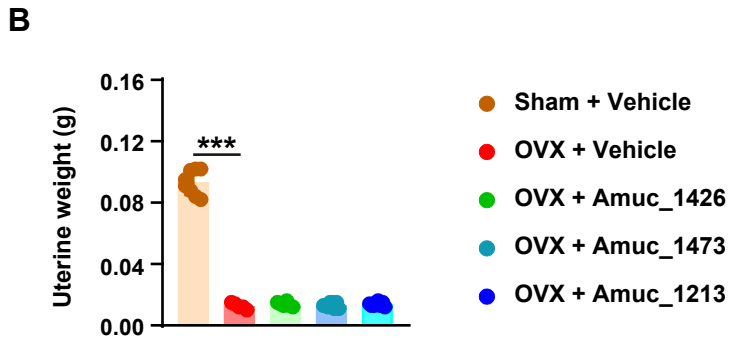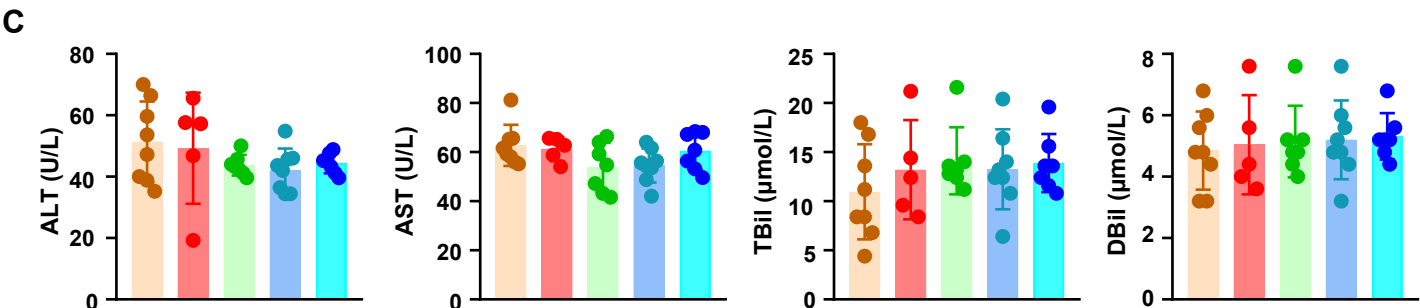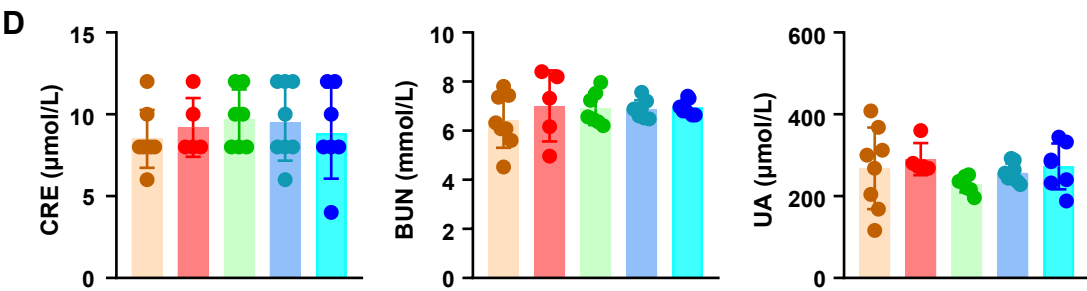

Supplement: Supplementary file 12 — Supporting File 12: advs75639‐sup‐0002‐Figure_S2.pdf. [file ADVS-9999-e23067-s008.pdf]

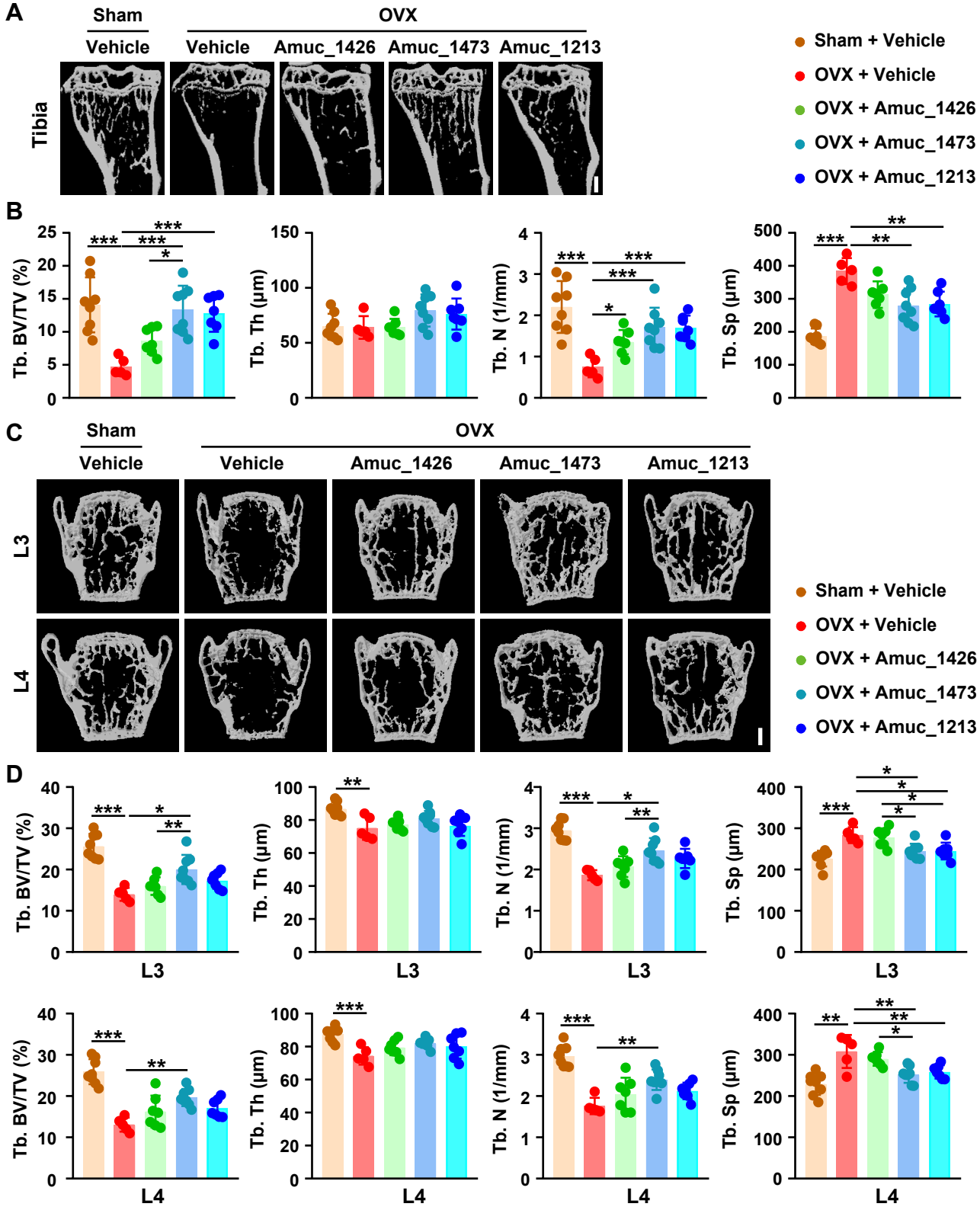

Supplement: Supplementary file 13 — Supporting File 13: advs75639‐sup‐0003‐Figure_S3.pdf. [file ADVS-9999-e23067-s005.pdf]

**A**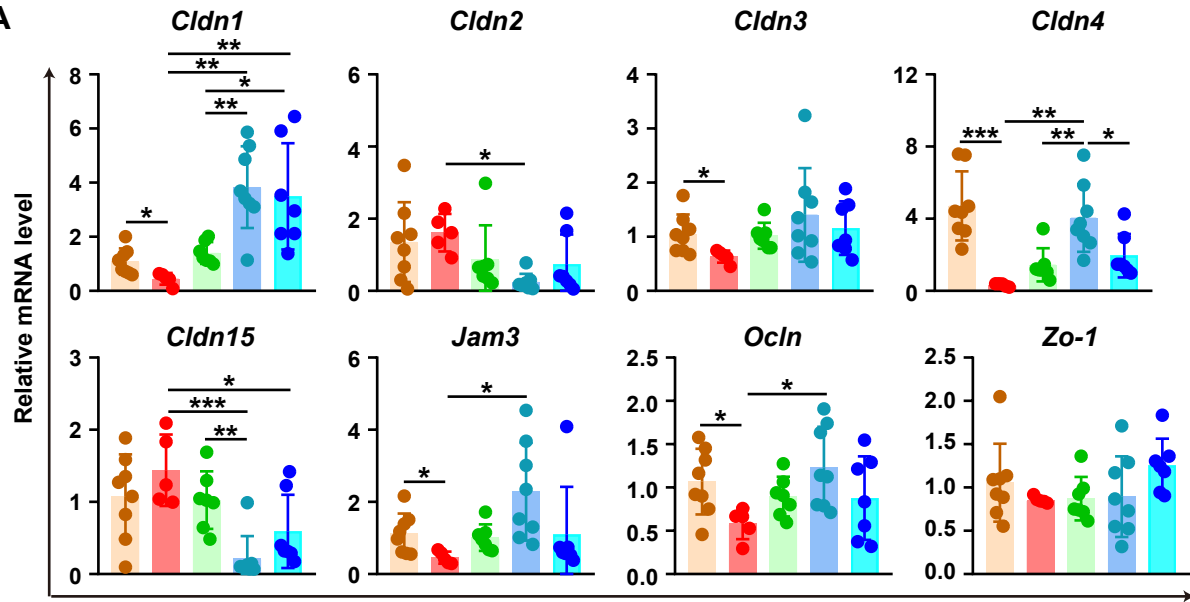

● Sham + Vehicle    ● OVX + Vehicle    ● OVX + Amuc\_1426    ● OVX + Amuc\_1473    ● OVX + Amuc\_1213

**B**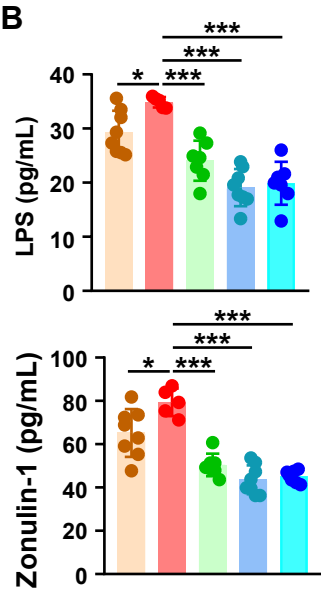

Supplement: Supplementary file 14 — Supporting File 14: advs75639‐sup‐0004‐Figure_S4.pdf. [file ADVS-9999-e23067-s017.pdf]

**A**

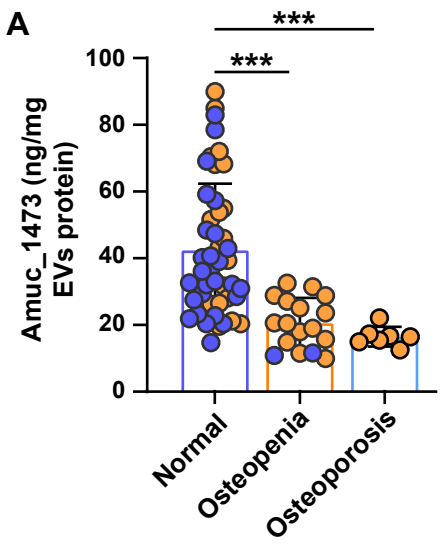

**B**

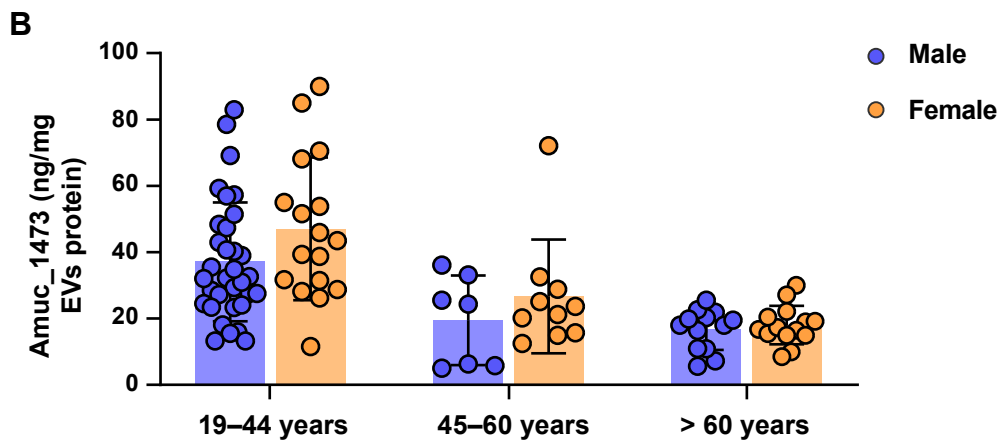

Supplement: Supplementary file 15 — Supporting File 15 advs75639‐sup‐0005‐Figure_S5.pdf. [file ADVS-9999-e23067-s003.pdf]

**A**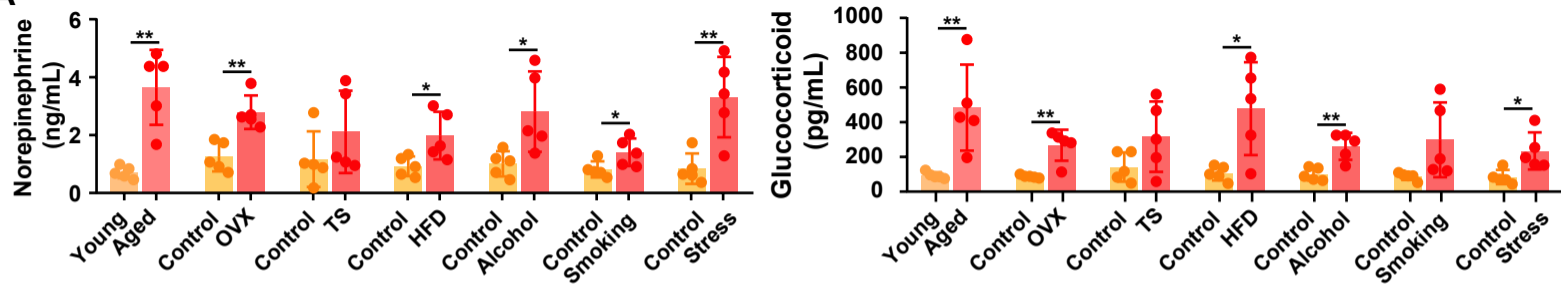**B**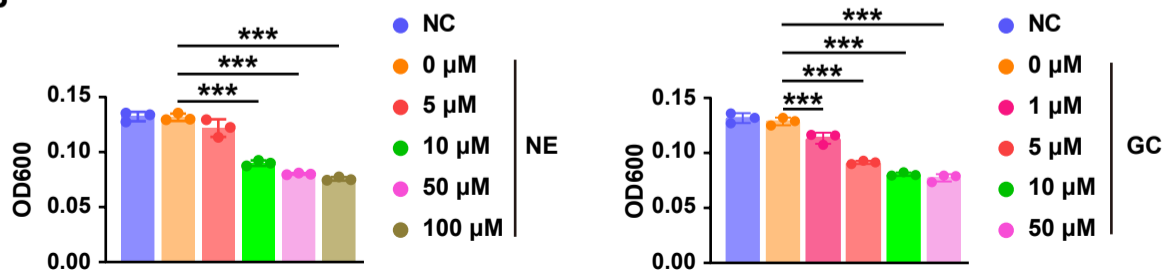

Supplement: Supplementary file 18 — Supporting File 18: advs75639‐sup‐0008‐Figure_S8.pdf. [file ADVS-9999-e23067-s006.pdf]
