# Supplementary material for: Amuc_1473 Links Gut Microbes to Skeletal Homeostasis and Counteracts Multifactorial Osteoporosis
Source: Adv Sci (Weinh). 2026 Jun 13:e23067. Online ahead of print. doi: 10.1002/advs.202523067 (PMC13335810; doi:10.1002/advs.202523067)
Supplement: Supplementary file 17 — Supporting File 17: advs75639‐sup‐0007‐Figure_S7.pdf. [file ADVS-9999-e23067-s015.pdf]

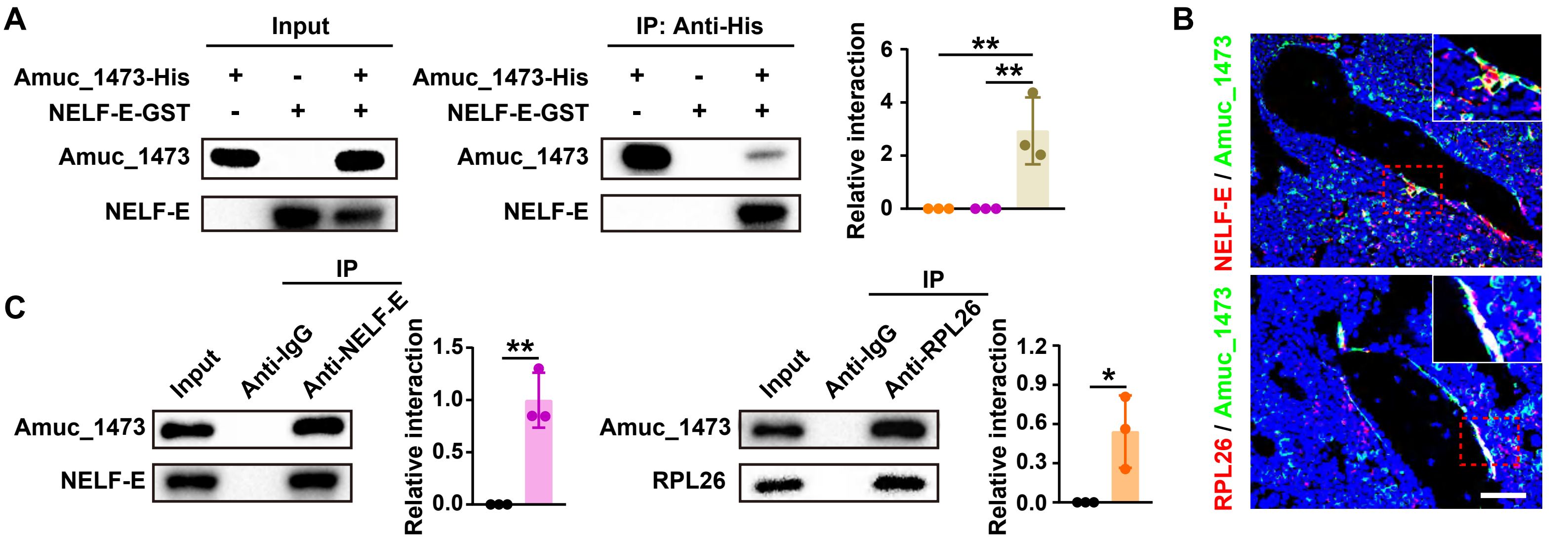
A
Input
IP: Anti-His
Amuc_1473-His
NELF-E-GST
Amuc_1473
NELF-E
Relative interaction
**
B
NELF-E / Amuc_1473
RPL26 / Amuc_1473
C
IP
Input
Anti-IgG
Anti-NELF-E
Anti-RPL26
RPL26
*
